# Artificial intelligence for good health: a scoping review of the ethics literature

**DOI:** 10.1186/s12910-021-00577-8

**Published:** 2021-02-15

**Authors:** Kathleen Murphy, Erica Di Ruggiero, Ross Upshur, Donald J. Willison, Neha Malhotra, Jia Ce Cai, Nakul Malhotra, Vincci Lui, Jennifer Gibson

**Affiliations:** 1 Joint Centre for Bioethics, Dalla Lana School of Public Health, University of Toronto, 155 College Street, Suite 754, Toronto, ON M5T 1P8 Canada; 2 Office of Global Health Education and Training, Dalla Lana School of Public Health, University of Toronto, 155 College Street, Room 408, Toronto, ON M5T 3M7 Canada; 3 Division of Clinical Public Health, Dalla Lana School of Public Health, 155 College Street, Toronto, ON M5T 3M7 Canada; 4 Bridgepoint Collaboratory for Research and Innovation, Lunenfeld Tanenbaum Research Institute, Sinai Health System, 1 Bridgepoint Drive, Toronto, ON M4M 2B5 Canada; 5 Institute of Health Policy, Management and Evaluation, Dalla Lana School of Public, Health Sciences Building, Health University of Toronto, 155 College Street, Suite 425, Toronto, ON M5T 3M6 Canada; 6 Gerstein Science Information Centre, University of Toronto, 9 King’s College Circle, Toronto, ON M7A 1A5 Canada

**Keywords:** Artificial intelligence, Ethics, Health care, Public and population health, Global health

## Abstract

**Background:**

Artificial intelligence (AI) has been described as the “fourth industrial revolution” with transformative and global implications, including in healthcare, public health, and global health. AI approaches hold promise for improving health systems worldwide, as well as individual and population health outcomes. While AI may have potential for advancing health equity within and between countries, we must consider the ethical implications of its deployment in order to mitigate its potential harms, particularly for the most vulnerable. This scoping review addresses the following question: What ethical issues have been identified in relation to AI in the field of health, including from a global health perspective?

**Methods:**

Eight electronic databases were searched for peer reviewed and grey literature published before April 2018 using the concepts of health, ethics, and AI, and their related terms. Records were independently screened by two reviewers and were included if they reported on AI in relation to health and ethics and were written in the English language. Data was charted on a piloted data charting form, and a descriptive and thematic analysis was performed.

**Results:**

Upon reviewing 12,722 articles, 103 met the predetermined inclusion criteria. The literature was primarily focused on the ethics of AI in health care, particularly on carer robots, diagnostics, and precision medicine, but was largely silent on ethics of AI in public and population health. The literature highlighted a number of common ethical concerns related to privacy, trust, accountability and responsibility, and bias. Largely missing from the literature was the ethics of AI in global health, particularly in the context of low- and middle-income countries (LMICs).

**Conclusions:**

The ethical issues surrounding AI in the field of health are both vast and complex. While AI holds the potential to improve health and health systems, our analysis suggests that its introduction should be approached with cautious optimism. The dearth of literature on the ethics of AI within LMICs, as well as in public health, also points to a critical need for further research into the ethical implications of AI within both global and public health, to ensure that its development and implementation is ethical for everyone, everywhere.

## Introduction

### Rationale

Artificial intelligence (AI) has been described as the “fourth industrial revolution” with transformative and global implications [1]. AI can be generally understood as “a field of study that combines computer science, engineering and related disciplines to build machines capable of behaviour that would be said to require intelligence were it to be observed in humans” [2]. Some such behaviours include the ability to visually perceive images, recognize speech, translate language, and learn from and adapt to new information [2]. To do so, AI as a field of study can employ a number of techniques. Machine learning, for instance, allows algorithms to make predictions and solve problems based on large amounts of data, without being explicitly programmed [2]. Deep learning is a subset of machine learning, and goes further to use multiple layers of artificial neural networks to solve complex problems from unstructured data, much like the human brain [2–4]. Many countries have developed or are in the process of developing national AI strategies and policies to promote research, development, and adoption of AI methods and technologies [5]. Amongst them, Canada was the first country to release a $125 million Pan-Canadian Artificial Intelligence Strategy to advance new public and private sector collaborations to stimulate research in AI [6]. Investments in AI are rapidly increasing with the potential for economic gains, projected at a $15.7 trillion contribution to the global economy by 2030 [7].

Amidst the nascence of AI, ethics has been identified as a priority concern in the development and deployment of AI across sectors [8–10]. In efforts to address this concern, there has been a proliferation of initiatives, including the establishment of organizations and principles documents [11] to provide guidance to those working within the AI space. Some such initiatives include the Partnership on AI [12], OpenAI [13], the Foundation for Responsible Robotics [14], the Ethics and Governance of Artificial Intelligence Initiative [15], the Montréal Declaration for Responsible Development of Artificial Intelligence [16], and the Principles for Accountable Algorithms [17, 18]. While there is increasing support from funding bodies for research on the social and ethical implications of AI [19–22], to date there has been limited attention by the academic bioethics community on AI within the field of health, particularly within the context of a globalized world. The health sector, however, is a growing area of AI research, development and deployment, with AI holding promise for the promotion of healthy behaviours; the detection and early intervention of infectious illnesses and environmental health threats; and the prevention, diagnosis, and treatment of disease [23–25].

The World Health Organization (WHO), for example, has established the “triple billion” target whereby it aims to have 1 billion more people benefit from universal health coverage, be better protected from health emergencies, and experience better health and wellbeing, and it believes that AI can help it achieve those objectives [26]. The WHO has been advancing the discussion of AI within health through its various Collaborating Centres, the AI for Global Good Summit, the development of the WHO Guideline Recommendations on Digital Interventions for Health System Strengthening [27], and its commitment to supporting countries in realizing the benefits of AI for health. Indeed, AI has been described by former WHO Director General Dr. Margaret Chan as the new frontier for health with transformative implications [28]. Yet amidst its promise, the introduction of AI in all corners of the world is accompanied by ethical questions that need to be uncovered from a global health perspective in order to be adequately addressed.

Global health has been defined as “an area for study, research, and practice that places a priority on improving health and achieving equity in health for all people worldwide” (p.1995), placing particular emphasis on the prevention and treatment of transnational population- and individual-level health issues through interdisciplinary and international collaboration [29]. To the extent that public health concerns the health of populations, global health concerns the health of populations on a global scale that transcends national boundaries and that underpins the interdependencies and interconnectivity of all people within a broader geopolitical, economic, and environmental context [29]. While both are critically important, AI, with its potential impact on research and development, trade, warfare, food systems, education, climate change, and more [30, 31], all of which either directly or indirectly impact the health of individuals, is inherently global.

In 2015, the 17 Sustainable Development Goals (SDGs) were unanimously adopted by all United Nations’ Member States. Goal 3 aims to achieve “good health and well-being” [32] and Goal 10 targets the reduction of inequalities [33]. While the SDGs are founded on the values of equity, inclusion, global solidarity, and a pledge to leave no one behind [34], the advent of AI could further exacerbate existing patterns of health inequities if the benefits of AI primarily support populations in high-income countries (HICs), or privilege the wealthiest within countries. Vinuesa and colleagues [35] assessed the role of AI in achieving all 17 SDGs (and their 169 targets), and found that while AI may serve predominantly as an enabler for achieving all targets in SDG 3, for SDG 10, it can be almost equally inhibiting as it is enabling. Considering, for instance, that many low- and middle-income countries (LMICs) still face significant challenges in digitizing their health records [36], data from which AI relies, there remains a substantial technological gap to overcome in order for LMICs to harness the potential benefits offered by AI. With increasing scale and diffusion of AI technologies in health worldwide, it is therefore imperative to identify and address the ethical issues systematically in order to realize the potential benefits of AI, and mitigate its potential harms, especially for the most vulnerable.

### Objectives

With this pursuit in mind, the purpose of this scoping review was to scope the academic and grey literatures in this emerging field, to better understand the discourse around the ethics of AI in health, and identify where gaps in the literature exist. Our research question was as follows: *What ethical issues have been identified in relation to AI in the field of health, including from a global health perspective?* Results from this scoping review of the academic and grey literatures include: (a) the selection of sources of evidence, (b) a descriptive analysis of the literature reviewed, (c) common ethical issues related to AI technologies in health, (d) ethical issues identified for specific AI applications in health, and (e) gaps in the literature pertaining to health, AI, and ethics.

## Methods

Our approach to scoping the literature was informed by the methods outlined by Levac, Colquhoun, and O’Brien [37], and the reporting guidelines established by Tricco, Lillie, Zarin, O'Brien, Colquhoun, Levac, et al. [38]. The core search concepts for the scoping review were AI, health, and ethics. Given the evolving nature of the AI field, both academic and grey literatures were included in the search. To enhance the rigour of our grey literature search specifically, the grey literature search was informed by search methods outlined by Godin, Stapleton, Kirkpatrick, Hanning, and Leatherdale [39].

### Eligibility criteria

**Fig. 1:**
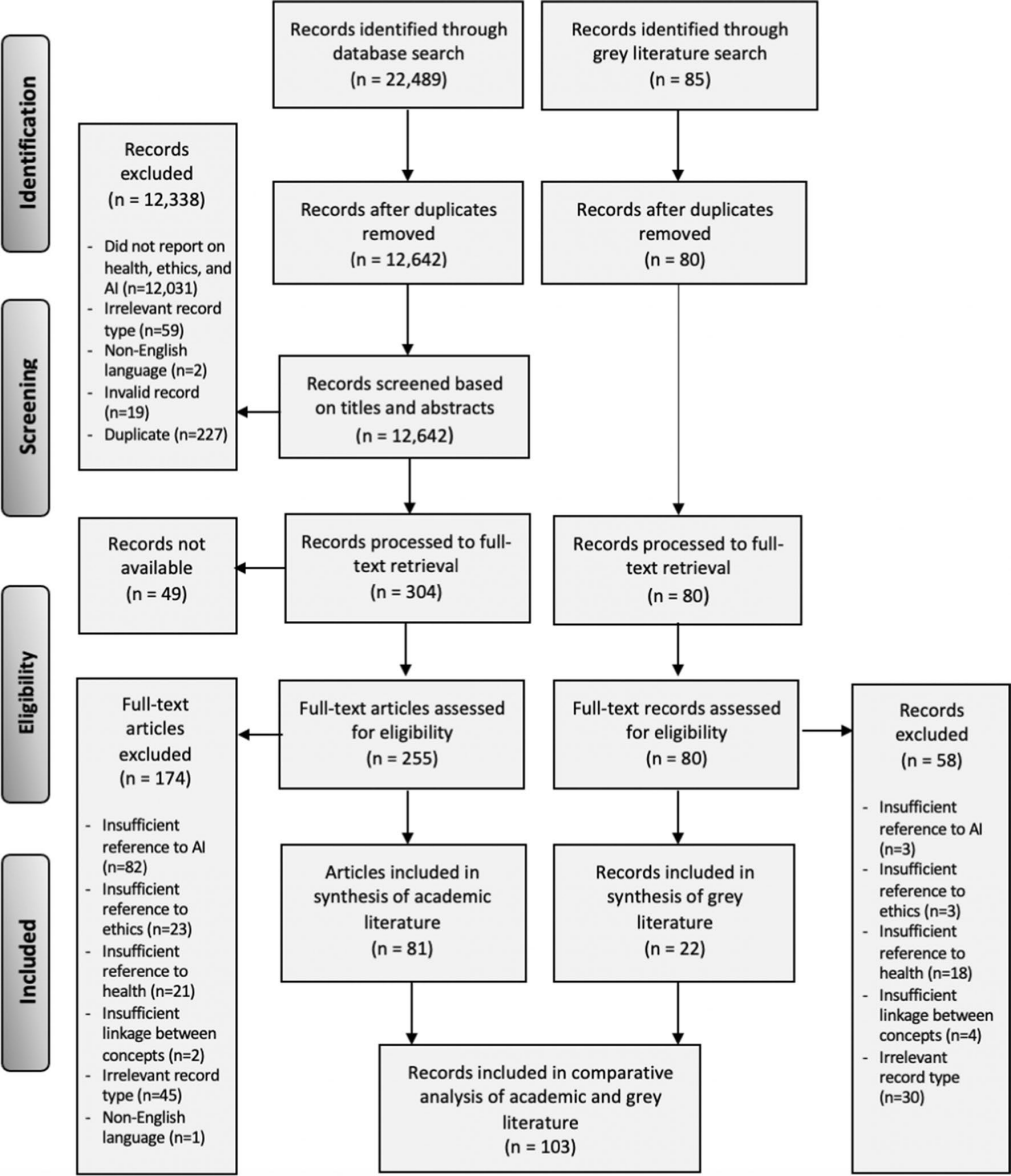
Preferred Reporting Items for Systematic Review and Meta-Analysis (PRISMA) flow diagram. This PRISMA flow diagram depicts the number of records identified at each state of the scoping review literature selection process

In keeping with a scoping review methodological approach [37], the inclusion and exclusion criteria were defined a priori and were refined as necessary throughout the iterative screening process involving the full project team at the beginning, middle, and end of the screening process to ensure consistency. Articles were selected during title and abstract screening if they met the following inclusion criteria: [1] records reported on all three core search concepts (AI, ethics, and health), and [2] records were written in the English language. The criterion for articles written in the English language was included because it is the language spoken by the majority of the research team, and thus allowed us to engage in a collaborative analysis process and enhance the rigour of our review. With regard to exclusion criteria, we excluded articles that did not include each of the concepts of AI, ethics and health, as well as those not written in the English language. Although ‘big data’ is a critical input to AI systems, articles that focused only on ethics and big data without explicit mention of AI methods or applications were excluded. Non-peer-reviewed academic literature was also excluded (e.g. letters, and non-peer reviewed conference proceedings), as were books and book chapters, each of which are categorized as ‘irrelevant record type’ in Fig. 1. Finally, invalid records (e.g. those that only included a string of code, or a date and no other information) and additional duplicates identified through the title/abstract screening process were excluded as well. No date or study design limits were applied, in order to obtain as extensive a literature base as possible. For the grey literature specifically, media articles, blog posts, and magazine entries were excluded, as we were more interested in documents that were both expert-driven, and which required a degree of methodological rigour (e.g. organization/institution reports). During full-text screening, records were excluded if any of the core search concepts were not engaged in a substantive way (e.g. if a concept was mentioned in passing or treated superficially); if there was an insufficient link made between health, ethics, and AI; if the ethics of AI was not discussed in relation to human health; if the article was not written in the English language; and if it was an irrelevant record type (e.g. a book, news article, etc.).

### Information sources

Searches of the peer-reviewed literature were executed in eight electronic databases: OVID MEDLINE (1946-present,includinge-pubaheadofprintandin-processandotherunindexedcitations), OVID Embase, (1947-present), OVID PsycINFO (1806-present), EBSCO CINAHL Plus with Full Text (1937-present), ProQuest Sociological Abstracts (1952-present), ProQuest Philosopher’s Index (1940-present), ProQuest Advanced Technologies & Aerospace (1962-present) and Wiley Cochrane Library. The search strategy was translated into each database using combinations of each database platform's command language, controlled vocabulary, and appropriate search fields, using MeSH terms, EMTREE terms, APA’s Thesaurus of Psychological Index Terms, CINAHL headings, Sociological Thesaurus, Philosopher’s Index subject headings, and Advanced Technologies & Aerospace subject headings in conjunction with keywords. Limits imposed were for English language-only articles; a filter excluding animal studies was applied to searches in MEDLINE, Embase, and PsycINFO, as we were interested in the ethics of AI as it applies to humans; and a filter for health or medicine-related studies was applied to the Advanced Technologies & Aerospace database, to reduce the high volume of solely technical studies. Final searches of the peer-reviewed literature were completed on April 23, 2018.

Grey literature was retrieved between April 25^th^ and September 12^th^, 2018, from (a) searches of grey literature databases including OAIster, Google Scholar, the Canadian Electronic Library, and the Canadian Institute for Health Information; (b) a Google search and customized Google search engines which included documents from think tanks, the Canadian government, and non-governmental organizations; (c) 28 targeted website searches of known organizations and institutions; and (d) the results from a prior environmental scan conducted by a member of the project team (J.G.). The targeted website searches were undertaken to identify any grey literature that was not captured in the grey literature databases and customized Google searches. The 28 websites searched were chosen based on the existing knowledge of members of the research team, in addition to input from stakeholders who attended an AI and health symposium in June 2018. For the purposes of feasibility and relevance, only reports from the year 2015 and beyond were retrieved.

### Search

The search strategy for the academic literature was developed by an academic health science librarian (V.L.) based on recommendations from the project leads (J.G., E.DiR., R.U.), and peer-reviewed by a second librarian. The full electronic search of the peer-reviewed literature can be found in Additional file 1, with an example search from OVID MEDLINE (1946-present,includinge-pubaheadofprintandin-processandotherunindexedcitations). The search strategy and results for the grey literature is similarly outlined in Additional file 2.

### Selection and sources of evidence

All identified records from the academic and grey literature searches were imported into the reference management software EndNote. After removing duplicate records, screening was conducted in two steps. First, the titles and abstracts of academic records were independently screened by two reviewers based on the inclusion and exclusion criteria established a priori. Reviewers consulted academic record keywords if the title and abstract lacked clarity in relation to the core concepts. Given that the majority of the grey literature did not include abstracts, grey literature records were screened initially on title. So as not to overlook relevant grey literature (given that some grey literature discussed ethical issues of AI more generally, including those pertaining to health), records proceeded to a full-text screening even if the title alluded to two of our three search concepts. A third reviewer assessed any records for which there was uncertainty among the reviewers about fit with the inclusion/exclusion criteria or discrepancy in reviewer assessments, and a final decision was made upon consensus with the research team. All records that passed the first level screening were pulled for full-text review by the two independent reviewers. The independent review and iterative team process were applied. The resulting sample was retained for data charting and analysis.

### Data charting process

Draft data charting forms for recording extracted data from the screened articles were created using Microsoft Excel (Version 16.18.(181,014)) based on the scoping review research question. As per the recommendations of Levac et al. [37], the data charting forms were piloted by having two project team members independently chart the first 10 academic and grey literature records [20 in total], with any arising discrepancies or uncertainties being brought to the larger project team for an agreed-upon resolution. The forms were further refined based on discussions with the project team and finalized upon consensus prior to completing the data charting process. For the remaining articles, each record was charted by one member of the research team, and weekly check-in meetings with the research team were held to ensure consistency in data charting, and to verify accuracy.

### Data items

We extracted data on the objective of each paper; the institutional affiliations of authors; the publication year; the country of the first and corresponding authors; whether a conflict of interest was stated; the health context of interest; the AI applications or technologies discussed; the ethical concepts, issues or implications raised; any reference to global health; and recommendations for future research, policy, or practice. Data was copy and pasted directly into the data charting form with the corresponding page number, so that no information was lost to paraphrasing. A template of the data charting form can be found in Additional file 3.

### Synthesis of results

The analysis comprised two components: descriptive and thematic. The descriptive analysis captured information about global location of primary authorship, dates of publication, and the AI application(s) discussed. Primary authorship was determined by the institutional location of the first author. The academic and grey literatures were compared to identify any notable differences in scope and emphasis. The thematic analysis [40] was conducted inductively. First, open descriptive codes were generated from a random sample of 10 academic records, and 10 grey literature records from which data had been extracted in the data charting form. Upon generating consensus among project team members on the appropriate codes after several attempts at refinement, codes were applied to meaningful data points throughout the entirety of the grey and academic records in the respective data charting forms, with new codes added as necessary. These codes were reorganized into themes and then compared amongst one another to identify commonalities and gaps in the literature, including convergences and divergences between the grey and academic literatures in relation to the original research question. Results are presented below in a narrative format, with complimentary tables and figures to provide visual representation of key findings.

## Results

### Selection of sources of evidence

Of the 12,722 records identified after de-duplication, 81 peer-reviewed articles and 22 grey literature records met the inclusion criteria for a total of 103 records in the scoping review sample (Fig. 1).

### Synthesis of results

#### Descriptive analytics

**Fig. 2:**
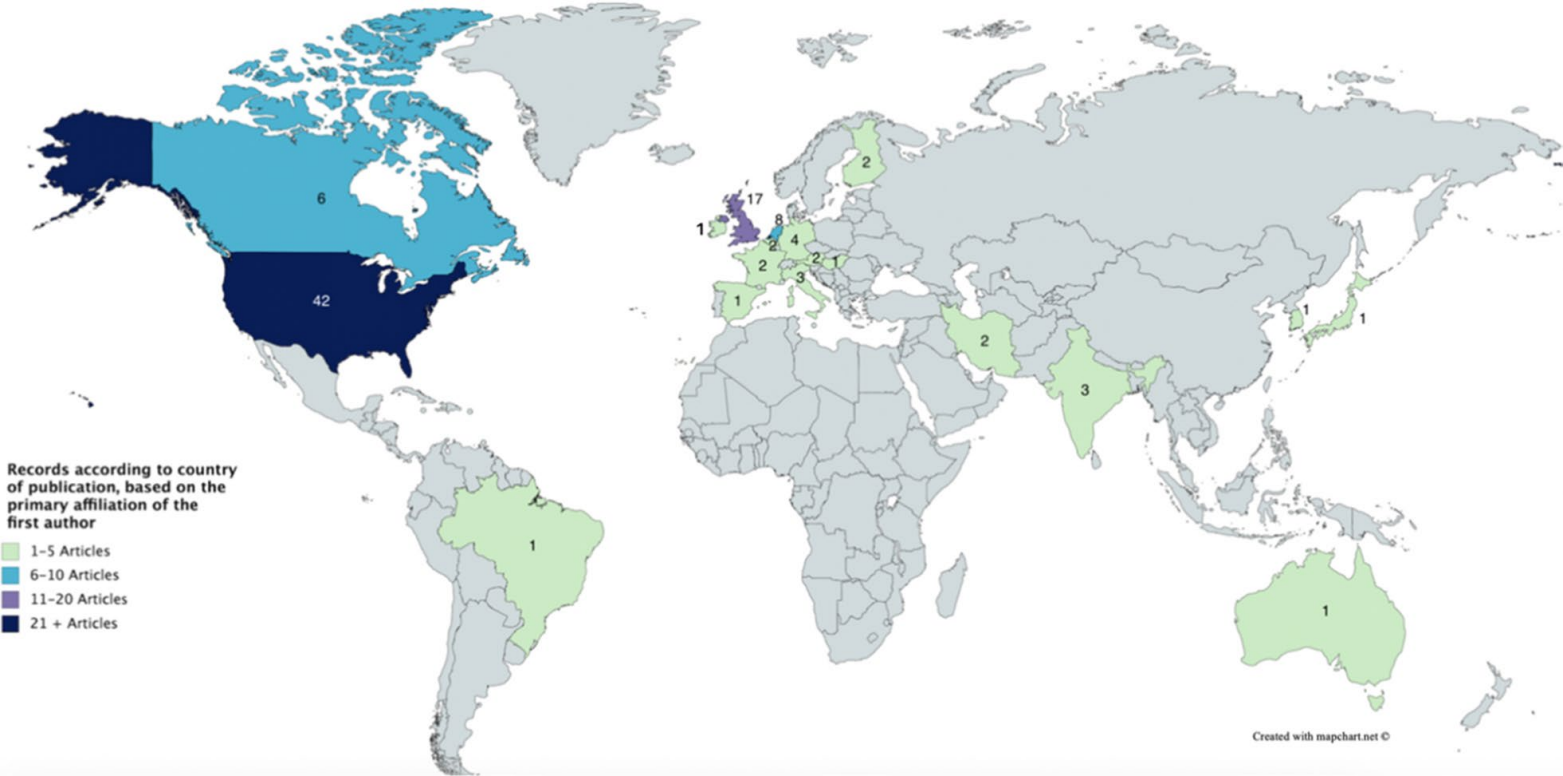
Number of publications by country, based on first author affiliation. *Note that two records were published by international organizations, and the geographic origin of one record is unknown. These three records are not represented in the above figure. This map was created using mapchart.net

**Fig. 3:**
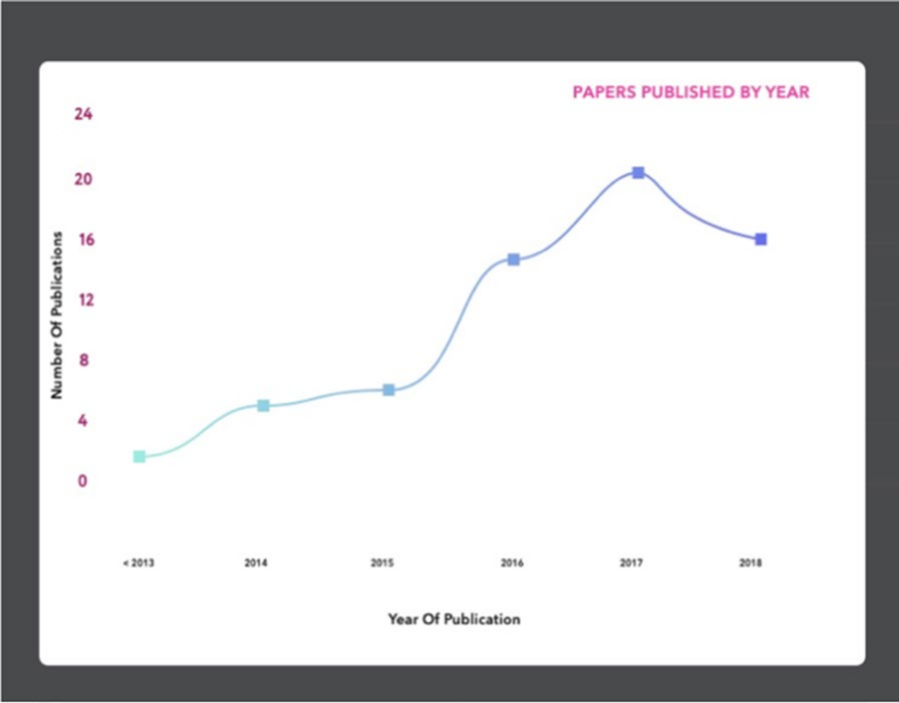
Number of publications reviewed, categorized by year of publication. *The graph begins in year 2013, after which the majority of articles were published

**Fig. 4:**
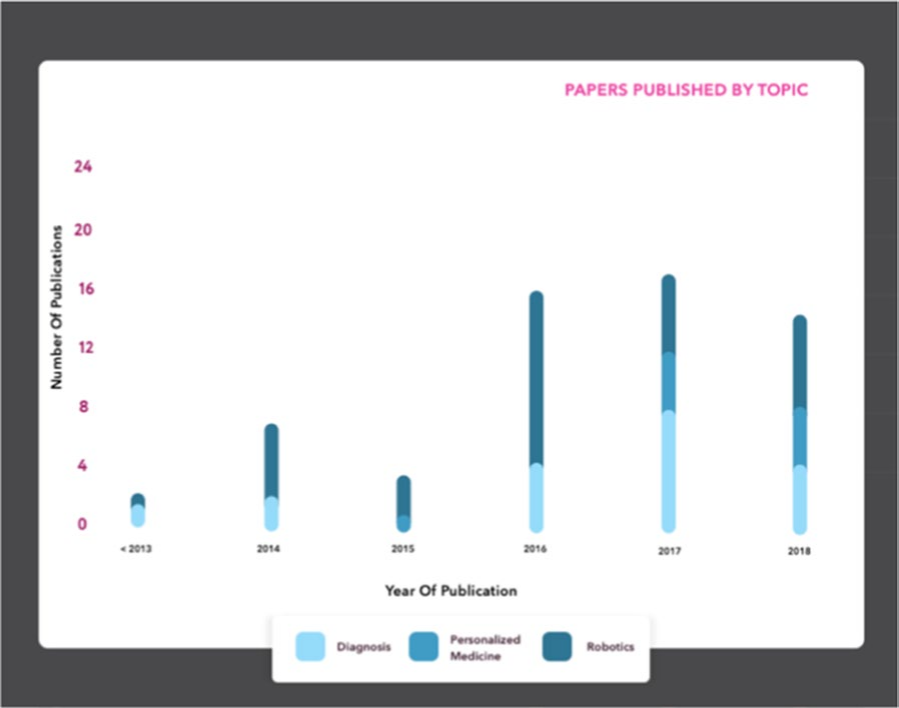
Publications reviewed according to the most frequently reported AI health applications. *The graph begins in year 2013, after which the majority of articles were published

The vast majority of publications had primary authors in the United States (n = 42) or the United Kingdom (n = 17) (Fig. 2) and while our literature search yielded publications between 1989 and 2018, most were published between 2014 and 2018 (Fig. 3). The academic and grey literatures addressed numerous AI-enabled health applications, including in particular, care robots^1^ (n = 48), followed by diagnostics (n = 36), and precision medicine (n = 16) (Fig. 4).

There were notable differences between the academic and grey literature sources in terms of authorship, AI health applications addressed, and treatment of ethical implications. The academic literature was written by persons primarily affiliated with academic institutions, whereas the grey literature was written by researchers, industry leaders, and government officials, often collaboratively, with authors frequently affiliated with multiple institutions. The grey literature tended to cover a broader range of AI health applications, issues, and trends, and their associated ethical implications, whereas the academic papers typically centered their discussion on one or at most a few topics or applications. The grey literature was oriented more towards broader health and social policy issues, whereas the academic literature tended to focus on a particular dimension of AI in health. As compared to the grey literature, robotics, particularly care robotics^(a)^ were highly represented in the peer-reviewed literature (48% of peer-reviewed literature, n = 39; 18% of the grey literature, n = 4). The academic literature on care robots was most concerned with the ethics of using care robots in health settings (e.g. “How much control, or autonomy, should an elderly person be allowed?”… “Are the safety and health gains great enough to justify the resulting restriction of the individual’s liberty?” (41, p.31, p.33), whereas the grey literature tended to emphasize ethical or operational implications of using robots in health settings, such as the potential displacement of human jobs [42].

#### Common ethical themes

Four ethical themes were common across the health applications of AI addressed in the literature, including data privacy and security, trust in AI, accountability and responsibility, and bias. These issues, while in many ways interconnected, were identified based on how distinctly they were discussed in the literature.

#### Privacy and security

Issues of privacy and data security were raised about the collection and use of patient data for AI-driven applications, given that these systems must be trained with a sizeable amount of personal health information [43, 44]. Highlighted concerns about the collection and use of patient data were that they may be used in ways unbeknownst to the individual from whom the information was collected [45], and that there is a potential for information collected by and for AI systems to be hacked [45]. One illustrative example of this challenge was that of the diagnostic laboratory database in Mumbai that was hacked in 2016, during which 35,000 patient medical records were leaked, inclusive of patient HIV status, with many patients never informed of the incident [45]. Further noted was that patients may believe that their data are being used for one purpose, yet it can be difficult to predict what the subsequent use may be [46, 47]. For example, ubiquitous surveillance for use by AI systems through personal devices, smart cities, or robotics, introduces the concern that granular data can be re-identified [48, 49], and personal health information can be hacked and shared for profit [49]. Of further concern was that these smart devices are often powered by software that is proprietary, and consequently less subject to scrutiny [48]. The stated implications of these privacy and security concerns were vast, with particular attention given to if ever personal data was leaked to employers and insurance companies [46, 50–54]. A prevailing concern was how population sub-groups may then be discriminated against based on their social, economic, and health statuses by those making employment and insurance decisions [49–51, 53].

### Trust in AI applications

The issues of privacy, security, and patient and healthcare professional [HCP] trust of AI were frequently and closely linked in the literature. Attention was given, for instance, to how individuals must be able to trust that their data is used safely, securely, and appropriately if AI technology is to be deployed ethically and effectively [2, 46, 55–57]. Asserted in the literature was that patients must be well enough informed of the use of their data in order to trust the technology and be able to consent or reject its use [52, 56]. One example that highlights these concerns is the data sharing partnership between Google DeepMind, an AI research company, and the Royal Free London NHS Foundation Trust (NHS) [49, 58]. Identifiable data from 1.6 million patients was shared with DeepMind with the stated intention of improving the management of acute kidney injuries with a clinical alert app [58]. However, there was a question of whether the quantity and content of the data shared was proportionate to what was necessary to test the app, and why it was necessary for DeepMind to retain the data indefinitely [49, 58]. Furthermore, this arrangement has come under question for being made in the absence of adequate patient consent, consultations with relevant regulatory bodies, or research approval, threatening patient privacy, and consequently public trust [49, 58].

HCPs have similarly demonstrated a mistrust in AI, resulting in a hesitancy to use the technology [59, 60]. This was exhibited, for instance, by physicians in various countries halting the uptake of IBM’s Watson Oncology, an AI-powered diagnostic support system [61]. These physicians stated that Watson’s recommendations were too narrowly focused on American studies and physician expertise, and failed to account for international knowledge and contexts [61]. The distrust amongst HCPs was also raised with regard to machine learning programs being difficult to both understand and explain [62, 63]. In contrast, a fear exists that some HCPs may place too much faith in the outputs of machine learning processes, even if the resulting reports, such as brain mapping results from AI systems, are inconclusive [57]. One suggestion to improve HCP trust in AI technology was to deploy training and education initiatives so HCPs have a greater understanding of how AI operates [43]. A further suggestion was to promote the inclusion of end-users in the design of the technology so that not only will end-users develop a better understanding of how it functions [64], but user trust will also increase through a more transparent development process [47].

#### Accountability and responsibility for use of AI technology

Frequently mentioned was the question of who ought to assume responsibility for errors in the application of AI technology to clinical and at-home care delivery [41, 45, 58–60, 65–67]. The question often arose in response to the fact that AI processes are often too complex for many individuals to understand and explain, which hinders their ability to scrutinize the output of AI systems [2, 61, 66]. Similarly, grounds for seeking redress for harm experienced as a result of its use were noted to be obstructed by the proprietary nature of AI technology, for under the ownership of private companies, the technology is less publicly accessible for inspection [2, 48, 51, 68]. Further to these questions, a debate remains as to whether or not HCPs ought to be held responsible for the errors of AI in the healthcare setting, particularly with regard to errors in diagnostic and treatment decisions [41, 45, 57, 65]. Several records put forward the view that, because HCPs are legally and professionally responsible for making decisions in their patient’s health interests, they bear responsibility for the consequences of decisions aided by AI technology [46, 47, 50, 59, 67, 69, 70]. However, records underlined the responsibility of manufacturers of AI systems for ensuring the quality of AI systems, including safety and effectiveness [47, 59, 71, 72], and for being responsive to the needs and characteristics of specific patient populations [72].

Beyond the clinical environment, issues of accountability arose in the context of using care robots. Related questions revolved around the burden of responsibility if an AI-enabled robotic care receiver is, for example, harmed by a robotic care provider [2, 73]. Is the burden of responsibility for such harm on the robot manufacturer who wrote the learning algorithm [73]? Similarly, the question arose of who is to be held accountable if a care receiver takes their own life or the life of another under the watch of a care robot [46]. If a care robot is considered an autonomous agent, should this incident then be the responsibility of the robot [46]? While proposed solutions to accountability challenges were few, one suggestion offered included building in a machine learning accountability mechanism into AI algorithms that could themselves perform black box audits to ensure they are privacy neutral (45, p.18). Also suggested was appropriate training of engineers and developers on issues of accountability, privacy, and ethics, and the introduction of national regulatory bodies to ensure AI systems have appropriate transparency and accountability mechanisms [45].

Where the above findings on accountability relate more to the “answerability” of AI’s potentially adverse impacts, responsibility was also present in the literature with regard to AI design and governance, albeit far less so. To promote responsible AI, governments were described as holding responsibility for developing policy to address ethical, social, and legal issues, including research and development of AI technologies, and for regulatory oversight [60, 74, 75]. Records also suggested that policymakers seek to understand public perceptions of the use of AI in health [75] and to ensure that AI technologies are distributed equally [74]. One article drew attention to the risk of exacerbating health inequities as a result of the unequal distribution of AI, particularly where AI applications are increasingly being used by patients for the self-management of their health [76]. While there was little mention of corporate responsibility, a small number of articles alluded to commercial strategies for responsible innovation [54, 55, 77]. Some such strategies included identifying where bias manifests and how and by whom it is managed; and being transparent in how the algorithm has been used (e.g. using a training dataset or in a real-world setting) and what type of learning the model is built for (e.g. supervised or unsupervised learning, etc.) [55]. Other suggestions included having AI manufacturing companies monitor the use of their systems in various contexts after being deployed [77], and to have AI research and development involve ‘human participation’ to ensure its conscientious development (54, p.10).

#### Adverse consequences of bias

Bias was yet another transcending ethical theme within the literature, notably the potential bias embedded within algorithms [43, 54, 59, 64, 68, 71, 77–79], and within the data used to train algorithms [43, 45, 49, 51, 55, 59–61, 63, 64, 68, 73, 77, 77, 78, 80–84]. The prevailing concern with algorithms was that they are developed by humans, who are by nature fallible, and subverted by their own values and implicit biases [68, 79]. These values have been noted to often reflect those that are societally endemic, and if carried into the design of AI algorithms, could consequently produce outputs that advantage certain population groups over others [43, 51, 54, 59, 63, 68, 71, 77, 77, 81]. Bias was indicated to similarly manifest in the data relied upon to train AI algorithms, by way of inaccurate and incomplete datasets [48, 51, 63, 81, 84], or by unrepresentative data sets [43, 82, 83], thus rendering AI outputs ungeneralizable to the population unto which it is applied [51, 68, 81].

Not only have biased data sets been noted to potentially perpetuate systemic inequities based on race, gender identity, and other demographic characteristics [48, 51, 59, 63, 68, 78], they may limit the performance of AI as a diagnostic and treatment tool due to the lack of generalizability highlighted above [43, 48, 83]. In contrast, some noted the potential for AI to mitigate existing bias within healthcare systems. Examples of this potential include reducing human error [50]; mitigating the cognitive biases of HCPs in determining treatment decisions, such as recency, anchoring, or availability biases [45, 51]; and reducing biases that may be present within healthcare research and public health databases [48]. Suggestions to address the issue of bias included building AI systems to reflect current ethical healthcare standards [78], and ensuring a multidisciplinary and participatory approach to AI design and deployment [79].

#### Specific ethical themes by AI application in health

Three health applications were emphasized in the reviewed literature: care robots, diagnostics, and precision medicine. Each health application raised unique ethical issues and considerations.

#### Care robotics

A notable concern for the use of care robots was the social isolation of care recipients, with care robots potentially replacing the provision of human care [41, 61, 72, 85–89]. Some asserted that the introduction of care robots would reduce the amount of human contact care recipients would receive from family, friends, and human care providers [41, 61, 72, 85, 87–89]. Implications of this included increased stress, higher likelihood of dementia, and other such impacts on the well-being of care recipients [41]. Others, in contrast, viewed robots as an opportunity to increase the “social” interaction that already isolated individuals may experience [41, 85, 90, 91]. Care robots could, for example, offer opportunities for care recipients to maintain interactive skills [91], and increase the amount of time human care providers spend having meaningful interactions with those they are caring for [85] as opposed to being preoccupied with routine tasks. Yet despite these opportunities, of note was the idea that care robots risk deceiving care recipients into having them believe that the robots are ‘real’ care providers and companions [41, 46, 72, 85, 87, 88, 92–94], which could undermine the preservation and promotion of human dignity [41, 92].

The issue of deception often linked to the question of ‘good care’, what the criteria for good care are, and whether robots are capable of providing it. In the context of deceit, some considered it justified as long as the care robot allows recipients to achieve and enhance their human capabilities [93, 95]. Also challenged was the assumption that good care is contingent upon humans providing it [46, 93, 96], for while robots may not be able to provide reciprocal emotional support [93], humans similarly may fail to do so [96]. A further illustrated aspect of good care was the preservation and advancement of human dignity [93], support for which can be offered by robots insofar as they promote individual autonomy [41, 61, 73, 85, 87, 88]. Some, however, contested this, arguing that care robots may in fact reduce a person’s autonomy if the technology is too difficult to use [87]; if the robot supersedes one’s right to make decisions based on calculations of what it thinks is best [61]; and because the implementation of robots may lead to the infantilization of care recipients, making them feel as though they are being treated like children [88]. The promotion of autonomy also appeared controversial, acknowledged at times as the pre-eminent value for which robots ought to promote [73, 91], where at others, autonomy was in tension with the safety of the care recipient [41, 91]. For example, with the introduction of care robots, care recipients might choose to engage in unsafe behaviours in pursuit of, and as a result of, their new independence [41, 91]. A comparable tension existed in the literature between the safety of care recipients, which some believe care robots protect, and the infringement on the recipient’s physical, and information privacy [41, 46, 88, 91, 97, 98].

### Diagnostics

Diagnostics was an area that also garnered significant attention with regard to ethics. Of note was the ‘black box’ nature of machine learning processes (36, 45, 51, 63, 74, 80, 99, 100), frequently mentioned with a HCP’s inability to scrutinize the output [44, 51, 63, 74]. Acknowledging that the more advanced the AI system, the more difficult it is to discern its functioning [99], there was also a concern that due to the difficulty in understanding how and why a machine learning program produces an output, there is a risk of encountering biased outputs [80]. Thus, despite the challenge of navigating these opaque AI systems, there was a call for said systems to be explainable in order to ensure responsible AI [45, 80]. Also a pervasive theme was the replacement and augmentation of the health workforce, particularly physicians, as a result of AI’s role in diagnostics [44, 59, 63, 100, 101]. While few feared the full replacement of physicians in diagnostics [2, 63, 100], some expected its presence to actually enhance the effectiveness and efficiency of their work [63, 100]. There were expressed concerns, however, about how the roles and interactions of physicians may change with its introduction, such as the ethical dilemma encountered if a machine learning algorithm is inconsistent with the HCP’s recommendation, if it contradicts a patient’s account of their own condition, or if it fails to consider patients’ non-verbal communication and social context [59].

#### Precision medicine

Issues of bias persisted in discussions of precision medicine, with the recognition that biased data sets, such as those that exclude certain patient populations, can produce inaccurate predictions that in turn can have unfair consequences for patients [81]. While precision medicine was a less prominent theme than the aforementioned AI applications, questions of the accuracy of predictive health information from the intersection of AI and genomics arose, as did an uncertainty of where and by whom that data may then be used [102]. In the case of AI-assisted gene editing, deep learning holds potential for directing experts where in the human genome to use gene editing technologies such as CRISPR, to reduce an individual’s risk of contracting a genetic disease or disorder [25]. However, deep learning models cannot discern the moral difference between gene editing for health optimization, and gene editing for human enhancement more generally, which may blur ethical lines [25]. A further tension existed in how the technology is deployed to support human choices; for example if a person not only seeks gene editing to reduce their risk of inheriting a particular genetic disease, but to also increase their muscle mass, obtain a particular personality trait, or enhance their musical ability [25]. Also illuminated was the implications of AI-enabled precision medicine in the global north versus the global south [103]. First is the possibility that this technology, given its high associated costs and greater accessibility in the developed world, might leave LMICs behind [103]. Second was the awareness that the introduction of genetic testing may undermine low cost, scalable and effective public health measures, which should remain central to global health [103].

#### Gaps in the literature

Healthcare was the predominant focus in the ethics literature on AI applications in health, with the ethics of AI in public health largely absent from the literature reviewed. One article that did illuminate ethical considerations for AI in public health highlighted the use of AI in environmental monitoring, motor vehicle crash prediction, fall detection, spatial profiling, and infectious disease outbreak detection, among other purposes, with the dominant ethical themes linking to data privacy, bias, and ‘black box’ machine learning models [82]. Other articles that mentioned public health similarly illustrated infectious disease outbreak predictions and monitoring [61, 84, 104], tracking communicable diseases [104], mental health research [105], and health behaviour promotion and management [59, 104]. However, these applications were only briefly mentioned in the broader context of primary healthcare, and few spoke to the ethics of these applications [59, 105, 106].

In the literature reviewed, there were also evident gaps in the area of global health, with few considerations of the unique ethical challenges AI poses for LMICs. Though there was mention of utilizing AI for screening in rural India [45]; genomics research in China [25]; facial recognition to detect malnutrition in Kenya [80]; and precision medicine in LMICs more broadly [103], among others, there was a significant gap in the literature commenting on the ethics of these practices in the global south. Furthermore, there was little discussion of health equity, including how the use of AI may perpetuate or exacerbate current gaps in health outcomes between and within countries. Instead, references to “global” health were often limited to global investments in AI research and development (R&D), and a number of innovations currently underway in HICs [25, 41, 49, 59, 73, 90, 107–109]. The lack of focus on global health was further reflected in the primary authorship of the literature, with a mere 5.8% (n = 6) of the reviewed literature authored by individuals from LMICs. Furthermore, 33% (n = 34) of articles had primary authorship from non-English speaking countries, which indicates that while the discourse of AI is indeed global in scope, it may only be reaching an Anglo-Saxon readership, or at the very least, an educated readership.

## Discussion

### Summary of evidence

#### Cross-cutting themes and asymmetries

In this scoping review we identified 103 records (81 academic articles and 22 grey literature articles) that addressed the ethics of AI within health, up to April 2018. Illustrated in the literature reviewed were overarching ethical concerns about privacy, trust, accountability, and bias, each of which were both interdependent and mutually reinforcing. Accountability, for instance, was a noted concern when considering who ought to bear responsibility for AI errors in patient diagnoses [63, 65, 66], while also a recognized issue in protecting patient privacy within data sharing partnerships [59]. The security of confidential patient data, in turn, was identified as critical for eliciting patient trust in the use of AI technology for health [2]. One suggestion offered to combat the threat to citizen trust in AI is through an inclusive development process [64], a process which has also been proposed to mitigate bias integrated into algorithm development [79]. It is therefore clear from our review that the aforementioned ethical themes cannot be considered in isolation, but rather must be viewed in relation to one another when considering the ethics of AI in health.

These broad ethical themes of privacy and security, accountability and responsibility, bias, and trust have also been revealed in other reviews. In a mapping review by Morley et al. [110] on AI in healthcare, for instance, concerns of trust, ‘traceability’ (aligning with what we have labelled ‘accountability’), and bias emerged. While privacy and security were explicitly excluded from their review [110], these very issues were a significant finding in a systematic review by Stahl et al. [111], both with regard to data privacy and personal (or physical) privacy. Issues of the autonomy and agency of AI machines, the challenge of trusting algorithms (linked with their lack of transparency), as well as others that were more closely associated with non-AI computing technologies were also discussed [111]. While the precise labels of ethical themes differed across these reviews based on the authors’ analytic approach, the general challenges were common across them, and indeed, intimately interconnected. It is clear also that these broad ethical themes are not unique to health, but rather transcend multiple sectors, including policing, transportation, military operations, media, and journalism [112, 113].

An asymmetry in the literature was the predominant focus on the ethics of AI in healthcare, with less attention granted to public health, including its core functions of health promotion, disease prevention, public health surveillance, and health system planning from a population health perspective. Yet in the age of ubiquitous computing, data privacy for use in public health surveillance and interventions will be all the more critical to secure, as will ensuring that individuals and communities without access to the latest technologies are not absent from these initiatives. In a recent article, Blasimme and Vayena [114] touched upon issues of consent when employing AI-driven social media analysis for digital epidemiology; the ethics of ‘nudging’ people towards healthier behaviours using AI technology; and developing paternalistic interventions tailored to marginalized populations. These public health issues and others merit further exploration within the ethics literature, particularly given how powerful such AI applications can be when applied at a population level. From an alternative perspective, the increasing presence of AI within healthcare may in some respects pose a risk to public health, with an expressed concern that the ‘hype’ around AI in healthcare may redirect attention and resources away from proven public health interventions [103, 115]. Similarly absent in the literature was a public health lens to the issues presented, a lens which rests on a foundation of social justice to “enable all people to lead fulfilling lives” [116]. With respect to jobs, for example, the pervasive discourse around care robots in the literature suggests that there may be a wave of robots soon to replace human caregivers of the sick, elderly, and disabled. Despite this recognition, however, the focus was solely on the impact on patients, and there was little mention given to those caregivers whose jobs may soon be threatened. This is true also for other low-wage workers within health systems at large, despite the fact that unemployment is frequently accompanied by adverse health effects.

A second asymmetry in the literature was the focus on HICs, and a notable gap in discourse at the intersection of ethics, AI, and health within LMICs. Some articles mentioned the challenges of implementing the technology in low-resource settings [25, 45, 80, 102, 103, 106], and whether its introduction will further widen the development gaps between HICs and LMICs [102], however absent in most was the integration of ethics and/or health. Yet AI is increasingly being deployed in the global south; to predict dengue fever hotspots in Malaysia [59], to predict birth asphyxia in LMICs at large [36], and to increase access to primary screening in remote communities in India [45], to name a few examples. Despite these advancements, in LMIC contexts there are challenges around collecting data from individuals without financial or geographic access to health services, data upon which AI systems rely [36, 80], and a further challenge of storing data electronically [80]. The United States Agency for International Development (USAID) and the Rockefeller Foundation [117] have recently illuminated some additional considerations for the deployment of AI in LMICs, one in particular being the hesitancy of governments and health practitioners to share digital health data for concern that it could be used against them, as digitizing health data is often quite politicized for actors on the ground. Given the infancy of these discussions, however, there is far more work to be done in order to critically and collaboratively examine the ethical implications of AI for health in all corners of the world, to ensure that AI contributes to improving, rather than exacerbating health and social inequities.

#### Towards ethical AI for health: what is needed?

Inclusive and participatory discourse and development of ethical AI for health was commonly recommended in the literature to mitigate bias [79], ensure the benefits of AI are shared widely [59, 74, 79, 80], and to increase citizens’ understanding and trust in the technology [47, 59, 64]. However, those leading the discussion on the ethics of AI in health seldom mentioned engagement with the end users and beneficiaries whose voices they were representing. While much attention was given to the impacts of AI health applications on underserved populations, only a handful of records actually included primary accounts from the people for whom they were raising concerns [2, 59, 75, 94, 118, 119]. Yet without better understanding the perspectives of end users, we risk confining the ethics discourse to the hypothetical, devoid of the realities of everyday life. This was illustrated, for instance, when participants in aged care challenged the ethical issue of care robots being considered deceptive, by stating that despite these concerns, they preferred a care robot over a human caregiver [94]. We therefore cannot rely on our predictions of the ethical challenges around AI in health without hearing from a broader mosaic of voices. In echoing recommendations from the literature, there is an evident need to gain greater clarity on public perceptions of AI applications for health, what ethical concerns end-users and beneficiaries have, and how best they can be addressed with the input of these individuals and communities. This recommendation is well aligned with the current discourse on the responsible innovation of AI, an important dimension of which involves the inclusion of new voices in discussions of the process and outcomes of AI [120].

In addition to taking a participatory approach to AI development, there is a responsibility for all parties to ensure its ethical deployment. For instance, it should be the responsibility of the producers of AI technology to advise end users, such as HCPs, as to the limits of its generalizability, just as should be done with any other diagnostic or similar technology. There is a similar responsibility for the end user to apply discretion with regards to the ethical and social implications of the technology they are using. This viewpoint is shared by Bonderman [121], who asserts that when physicians deploy AI during patient diagnoses, for instance, it is important that they remain in control, and retain the authority to override algorithms when they have certainty the algorithm outputs are incorrect [122]. Ahuja [122] compliments this assertion by stating how, since machine learning and deep learning require large quantities of data, said systems can underperform when presented with novel cases, such as atypical side effects or resistance to treatment. Simply stated, we must be critical and discretionary with regards to the application of AI in scenarios where human health and wellbeing are concerned, and we must not simply defer to AI outputs.

Also in need of critical reflection, as it remains unresolved in the literature, is how to appropriately and responsibly govern this technology [25, 45, 49, 52, 57, 102]. While there were hints in the literature regarding how to promote responsible AI, such as equal distribution of the technology, corporate transparency, and participatory development, there was little on how these recommendations could be optimally secured through regulatory mechanisms and infrastructure. The infusion of AI into health systems appears inevitable, and as such, we need to reconsider our existing regulatory frameworks for disruptive health technologies, and perhaps deliberate something new entirely. Given the challenge that many have termed the ‘black box’, illustrative of the fact that, on the one hand, AI processes operate at a level of complexity beyond the comprehension of many end-users, and on the other, neural networks are by nature opaque, the issue of governance is particularly salient. Never before has the world encountered technology that can learn from the information it is exposed to, and in theory, become entirely autonomous. Even the concept of AI is somewhat nebulous [2, 59, 123, 124], which threatens to cloud our ability to govern its use. These challenges are compounded by those of jurisdictional boundaries for AI governance, an ever-increasing issue given the global ‘race’ towards international leadership in AI development [125]. Thirty-eight national and international governing bodies have established or are developing AI strategies, with no two the same [125, 126]. Given that the pursuit of AI for development is a global endeavour, this calls for governance mechanisms that are global in scope. However, such mechanisms require careful consideration in order for countries to comply, especially considering differences in national data frameworks that pre-empt AI [49]. These types of jurisdictional differences will impact the ethical development of AI for health, and it is thus important that academic researchers contribute to the discussion on how a global governance mechanism can address ethical, legal, cultural, and regulatory discrepancies between countries involved in the AI race.

### Limitations

One potential limitation to this study is that given the field of AI is evolving at an unprecedented rate [1], there is a possibility that new records in the academic and grey literatures will have been published after the conclusion of our search, and prior to publication. Some recent examples of related articles have very much been in line with our findings, drawing light to many of the pertinent ethical issues of AI in healthcare discussed in the literature reviewed [18, 127–132]. Few, however, appear to have discussed the ethical application of AI in LMICs [117, 133] or public health [117, 130], so despite any new literature that may have arisen, there is still further work to be done in these areas. Furthermore, given our search strategy was limited to the English language, we may have missed valuable insights from publications written in other languages. The potential impact on our results is that we underrepresented the authorship from LMICs, and underreported the amount of literature on the ethics of AI within the context of LMICs. Furthermore, by not engaging with literature in other languages, we risk contradicting recommendations for an inclusive approach to the ethics discourse. Indeed, we may be missing important perspectives from a number of country and cultural contexts that could improve the ethical development and application of AI in health globally. To address this limitation, future researchers could collaborate with global partner organizations, such as WHO regional offices, in order to gain access to literatures which would otherwise be inaccessible to research teams. An additional limitation lies in our grey literature search. As part of a systematic search strategy, we pursued targeted website searches in order to identify any literature that did not emerge from our grey literature database and customized Google searches. These websites were chosen based on the expert knowledge of the research team, as well as stakeholders operating within the AI space, however there is a chance that additional relevant websites, and thus reports, proceedings, and other documents, exist beyond what was included in this review. Nevertheless, this scoping review offers a comprehensive overview of the current literature on the ethics of AI in health, from a global health perspective, and provides a valuable direction for further research at this intersection.

## Conclusions

The ethical issues surrounding the introduction of AI into health and health systems are both vast and complex. Issues of privacy and security, trust, bias, and accountability and responsibility have dominated the ethical discourse to date with regard to AI and health, and as this technology is increasingly taken to scale, there will undoubtedly be more that arise. This holds particularly true with the introduction of AI in public health, and within LMICs, given that these areas of study have been largely omitted from the ethics literature. AI is being developed and implemented worldwide, and without considering what it means for populations at large, and particularly those who are hardest to reach, we risk leaving behind those who are already the most underserved. Thus, the dearth of literature on the ethics of AI within public health and LMICs points to a critical need to devote further research in these areas. Indeed, a greater concentration of ethics research into AI and health is required for all of its many applications. AI has the potential to help actualize universal health coverage, reduce health, social, and economic inequities, and improve health outcomes on a global scale. However, the bourgeoning field of AI is outpacing our ability to adequately understand its implications, much less to regulate its responsible design, development, and use for health. Given the relatively uncharted territory of AI in health, we must be diligent to both consider and respond to the ethical implications of its implementation, and whether if in every case it is indeed ethical at all. Amidst the tremendous potential that AI carries, it is important to approach its introduction with a degree of cautious optimism, informed by an extensive body of ethics research, to ensure its development and implementation is ethical for everyone, everywhere.

## Supplementary Information

**Additional file 1.** Search Strategy for the Academic Literature.

**Additional file 2.** Search Strategy and Results of Grey Literature Search.

**Additional file 3.** Data Charting Form Template.

**Additional file 4.** Bibliography of the 103 records included in analysis.

## Data Availability

The datasets used and/or analysed during the current study are available from the corresponding author upon reasonable request.

## Abbreviations

AI: Artificial intelligence
CIHR: Canadian Institutes of Health Research
SDG(s): Sustainable Development Goal(s)
LMIC(s): Low- and middle-income country/ies
HIC(s): High-income country/ies
HCP(s): Health care professionals
R&D: Research and development
CRISPR: Clustered regularly interspaced short palindromic repeats

## Footnotes

1 Robots for the care of the sick, elderly, or disabled bore a number of different labels in the literature, however they will herein be described as ‘care robots’ in an effort to broadly discuss the associated ethical challenges. ‘Care robots’ as used in this context are exclusive of surgical robots. Only those care robots that relied on AI are discussed, such as those that can understand commands, can locate and pick up objects, relocate a patient, and other tasks that require machine intelligence.
