# Supplementary material for: Artificial intelligence for good health: a scoping review of the ethics literature
Source: BMC Med Ethics. 2021 Feb 15;22:14. doi: 10.1186/s12910-021-00577-8 (PMC7885243; doi:10.1186/s12910-021-00577-8)
Supplement: Supplementary file 1 — Additional file 1. Search Strategy for the Academic Literature. [file 12910_2021_577_MOESM1_ESM.docx]

*File Name:* **Additional File 1**

*File format:* Word document (.docx)

*Title of data:* **Search Strategy for the Academic Literature**

*Description of data:* Ovid MEDLINE: Epub Ahead of Print, In-Process & Other Non-Indexed Citations, Ovid MEDLINE® Daily and Ovid MEDLINE® <1946-Present>

*Note: this search strategy was translated into the other 7 academic databases using combinations of each database platform's command language, controlled vocabulary, and appropriate search fields, using MeSH terms, EMTREE terms, APA’s Thesaurus of Psychological Index Terms, CINAHL headings, Sociological Thesaurus, Philosopher’s Index subject headings, and Advanced Technologies & Aerospace subject headings in conjunction with keywords.

| **#** | **Searches** |
| --- | --- |
| 1 | artificial intelligence/ |
| 2 | Machine Learning/ |
| 3 | Computer Heuristics/ |
| 4 | Expert Systems/ |
| 5 | knowledge bases/ |
| 6 | Natural Language Processing/ |
| 7 | "neural networks (computer)"/ |
| 8 | Robotics/ |
| 9 | Decision Making, Computer-Assisted/ |
| 10 | Diagnosis, Computer-Assisted/ |
| 11 | Support Vector Machine/ |
| 12 | Pattern Recognition, Automated/ |
| 13 | supervised machine learning/ |
| 14 | unsupervised machine learning/ |
| 15 | cybernetics/ |
| 16 | data mining/ |
| 17 | Medical Informatics/ |
| 18 | Wearable Electronic Devices/ or Fitness trackers/ |
| 19 | social media/ and (IoT or "Internet of Things" or autom* or auton* or wearable* or (fitness adj2 (track* or app or apps or application*)) or virtual assistan* or IVA or IVAs or smart speaker* or (big adj2 data*)).tw,kf. |
| 20 | ((artificial or comput* or machine* or device*) adj4 intelligen*).tw,kf. |
| 21 | (Machine learning or machine-learning).tw,kf. |
| 22 | robot*.tw,kf. |
| 23 | (Deep learning or deep-learning).tw,kf. |
| 24 | (deep mind* or deep-mind* or deepmind*).tw,kf. |
| 25 | ((comput* or automat*) adj4 reasoning).tw,kf. |
| 26 | (data mining or data-mining or datamining).tw,kf. |
| 27 | (Super intelligen* or super-intelligen* or superintelligen*).tw,kf. |
| 28 | (telerobot* or tele-robot*).tw,kf. |
| 29 | (cogniti* adj2 comput*).tw,kf. |
| 30 | (natural language process* or NLP*).tw,kf. |
| 31 | (neural network* adj4 (model* or comput* or artificial or deep)).tw,kf. |
| 32 | perceptron*.tw,kf. |
| 33 | (comput* adj4 (decision mak* or decision-mak* or decisionmak*)).tw,kf. |
| 34 | (heuristic* adj2 (comput* or intelligen*)).tw,kf. |
| 35 | AICP*.tw,kf. |
| 36 | turing*.tw,kf. |
| 37 | naive bayes.tw,kf. |
| 38 | (bayesian adj4 learning).tw,kf. |
| 39 | ("k-nearest neighbor" or "k-nearest neighbour" or k-NN).tw,kf. |
| 40 | (Super comput* or super-comput* or supercomput*).tw,kf. |
| 41 | random forest*.tw,kf. |
| 42 | (cybernetic* or cyborg* or cyber medicine or cyber-medicine or cybermedicine).tw,kf. |
| 43 | (support vector machine* or support vector network* or SVM* or SVN*).tw,kf. |
| 44 | (big adj2 data*).tw,kf. |
| 45 | (predictive adj2 analy*).tw,kf. |
| 46 | (wearable* or (fitness adj2 (track* or app or apps or application*))).tw,kf. |
| 47 | (IoT or "internet of things").tw,kf. |
| 48 | (smart glass* or smart-glass* or smartglass* or Google glass* or Google Lens* or Google Goggles or HoloLens*).tw,kf. |
| 49 | ((Google or Amazon or Apple or Alexa or Siri or IBM* or Watson or Microsoft or Cortana or Facebook or Twitter or Instagram or social media or social network*) and (IoT or "Internet of Things" or autom* or auton* or wearable* or (fitness adj2 (track* or app or apps or application*)) or virtual assistan* or IVA or IVAs or smart speaker* or (big adj2 data*))).tw,kf. |
| 50 | or/1-49 |
| 51 | Ethics/ |
| 52 | Bioethical Issues/ |
| 53 | Bioethics/ |
| 54 | Ethics, Clinical/ |
| 55 | "Codes of Ethics"/ |
| 56 | complicity/ |
| 57 | "Conflict of Interest"/ |
| 58 | informed consent/ or consent forms/ or informed consent by minors/ or third-party consent/ |
| 59 | Ethical Analysis/ |
| 60 | Ethical Relativism/ |
| 61 | "Ethical Review"/ |
| 62 | Ethical Theory/ |
| 63 | Ethicists/ |
| 64 | Ethics Committees/ |
| 65 | Ethics, Business/ |
| 66 | Ethics, Institutional/ |
| 67 | Ethics, Professional/ |
| 68 | Ethics, Research/ |
| 69 | Humanism/ |
| 70 | moral status/ |
| 71 | personhood/ |
| 72 | principle-based ethics/ |
| 73 | Professional Misconduct/ |
| 74 | Morals/ |
| 75 | moral development/ |
| 76 | moral obligations/ |
| 77 | conscience/ |
| 78 | virtues/ |
| 79 | Social Responsibility/ |
| 80 | Human Rights/ |
| 81 | Civil Rights/ |
| 82 | freedom/ |
| 83 | Social Justice/ |
| 84 | Social Values/ |
| 85 | Philosophy, Medical/ |
| 86 | confidentiality/ |
| 87 | jurisprudence/ |
| 88 | disclosure/ |
| 89 | personally identifiable information/ |
| 90 | data anonymization/ |
| 91 | Clinical Governance/ |
| 92 | "Delivery of Health Care"/es or public health administration/es or public policy/es or public health administration/es [Ethics] |
| 93 | (ethic* or unethic* or bioethic*).tw,kf,jn. |
| 94 | consent.tw,kf. |
| 95 | equit*.tw,kf. |
| 96 | complicit*.tw,kf. |
| 97 | "Conflict* of interest*".tw,kf. |
| 98 | Humanism.tw,kf. |
| 99 | Moral*.tw,kf. |
| 100 | Personhood.tw,kf. |
| 101 | Conscience.tw,kf. |
| 102 | (Public good or common good or public interest*).tw,kf. |
| 103 | (fair or fairness).tw,kf. |
| 104 | (Justice or injustice*).tw,kf. |
| 105 | (Private or privacy).tw,kf. |
| 106 | Human right*.tw,kf. |
| 107 | freedom*.tw,kf. |
| 108 | Social justice*.tw,kf. |
| 109 | Social value*.tw,kf. |
| 110 | Civil right*.tw,kf. |
| 111 | philosoph*.tw,kf. |
| 112 | ((doctrine* or principle* or rule*) adj2 (double effect or double-effect)).tw,kf. |
| 113 | ((social* or societ* or moral*) adj4 (responsib* or obligat*)).tw,kf. |
| 114 | accountab*.tw,kf. |
| 115 | confidential*.tw,kf. |
| 116 | (Jurisprudence or law* or legal*).tw,kf. |
| 117 | disclos*.tw,kf. |
| 118 | (Data* adj2 anonym*).tw,kf. |
| 119 | Governance.tw,kf. |
| 120 | or/51-119 |
| 121 | 50 and 120 |
| 122 | ((roboethic* or robo-ethic* or (robot* or machine* or artificial)) adj4 (ethic* or bioethic* or unethic*)).tw,kf. |
| 123 | 121 or 122 |
| 124 | degree* of freedom*.tw,kf. |
| 125 | 123 not 124 |
| 126 | exp Animals/ not (Humans/ and exp Animals/) |
| 127 | 125 not 126 |
| 128 | limit 127 to english language |
