# Supplementary material for: Artificial intelligence for good health: a scoping review of the ethics literature
Source: BMC Med Ethics. 2021 Feb 15;22:14. doi: 10.1186/s12910-021-00577-8 (PMC7885243; doi:10.1186/s12910-021-00577-8)
Supplement: Supplementary file 2 — Additional file 2. Search Strategy and Results of Grey Literature Search. [file 12910_2021_577_MOESM2_ESM.docx]

*File name:* **Additional File 2**

*File format:* Word document (.docx)

*Title of data:* **Search Strategy and Results of Grey Literature Search**

*Description of data:* Search Strategy and Results of Grey Literature Search

**Grey Literature Databases** (May 10, 2018)

Total # Records before de-duplication: 22

Total # Records after de-duplication: 21

| Database | Search Strategy | # Records Screened | # New Potentially Relevant Records (after de | #Records after de-duplication |
| --- | --- | --- | --- | --- |
| Google Scholar | Artificial Intelligence and health and ethics | 50 | 20 | 19 |
| Des Lebris/Canadian Electronic Library | Artificial Intelligence | 4 | 0 | 0 |
| Canadian Institute for Health Information | Artificial Intelligence | 0 | 0 | 0 |
|  | Machine Learning | 5 | 0 | 0 |
| OAIster | Artificial Intelligence AND health AND ethics | 13 | 2 | 2 |

**Customized Google Search Engines** (May 13, 2018)

Total # Records before de-duplication: 26

Total # Records after de-duplication: 24

| Search Engine | Search Strategy | # Records Screened | | # New Potentially Relevant Records | #Records after de-duplication |
| --- | --- | --- | --- | --- | --- |
| Think Tanks | Artificial intelligence and health and ethics | 50 | | 7 | 7 |
| Non-Governmental Organizations | Artificial intelligence and health and ethics | 50 | | 0 | 0 |
| Canadian Government Documents | Artificial intelligence and health and ethics | Federal Documents | 0 | 0 | 0 |
|  |  | Provincial Documents | 0 | 0 | 0 |
|  |  | Municipal Documents | 0 | 0 | 0 |
|  |  | Depository Publications (Online Catalogue) | 4 | 2 | 2 |
| Google Search | Artificial intelligence and health and ethics | 45 | | 17 | 15 |

**Targeted Websites**

Total # Records before de-duplication: 37

Total # Records after de-duplication: 35

| Websites | URL | # Records Identified | Date Accessed | #Records after de-duplication |
| --- | --- | --- | --- | --- |
| MacArther Foundation | https://www.macfound.org/ | 0 | May 13, 2018 | 0 |
| Peterson Institute for International Economics | https://piie.com/ | 0 | May 13, 2018 | 0 |
| American Civil Liberties Union | https://www.aclu.org/ | 0 | May 13, 2018 | 0 |
| UC Berkeley | https://www.berkeley.edu/ | 0 | May 13, 2018 | 0 |
| Association for the Advancement of Artificial Intelligence at ASU | https://www.aaai.org/ | 0 | May 13, 2018 | 0 |
| OpenAI | https://openai.com/ | 0 | April 30, 2018 | 0 |
| Artificial Intelligence in Medicine Inc. | http://www.aim.ca/ | 0 | May 13, 2018 | 0 |
| AI Now Institute | https://ainowinstitute.org/ | 4 | May 13, 2018 | 4 |
| Mowat Centre | https://mowatcentre.ca/ | 0 | May 13, 2018 | 0 |
| Nuffield Council on Bioethics | http://nuffieldbioethics.org/ | 1 | April 25, 2018 | 1 |
| European Data Protection Supervisor - Ethics Advisory Group | https://royalsociety.org/~/media/policy/projects/data-governance/data-management-governance.pdf | 1 | April 25, 2018 | 1 |
| Stanford University | https://ai100.stanford.edu/2016-report | 1 | April 25, 2018 | 1 |
| The AI Initiative and The Future Society - Harvard Kennedy School | http://ai-initiative.org/wp-content/uploads/2017/08/Making-the-AI-Revolution-work-for-everyone.-Report-to-OECD.-MARCH-2017.pdf | 1 | April 25, 2018 | 1 |
| Centre for Internet and Society (CIS) | https://cis-india.org/internet-governance/files/ai-and-healtchare-report | 1 | April 25, 2018 | 1 |
| IEEE Global Initiative on Ethics of Autonomous and Intelligent Systems | https://standards.ieee.org/develop/indconn/ec/autonomous_systems.html | 1 | April 25, 2018 | 1 |
| The Alan Turing Institute | https://www.turing.ac.uk/ | 0 | May 14, 2018 | 0 |
| Canadian Institute for Advanced Research | https://www.cifar.ca/ai/ | 0 | May 14, 2018 | 0 |
| McKinsey | https://www.mckinsey.com/~/media/mckinsey/global%20themes/china/artificial%20intelligence%20implications%20for%20china/mgi-artificial-intelligence-implications-for-china.ashx | 1 | April 20, 2018 | 1 |
| Deloitte | https://www2.deloitte.com/insights/us/en/deloitte-review/issue-21/artificial-intelligence-and-the-future-of-work.html | 1 | May 14, 2018 | 1 |
| CBI | http://www.cbi.org.uk/about/about-us/ | 0 | May 14, 2018 | 0 |
| Vector Institute for Artificial Intelligence | https://vectorinstitute.ai/ | 0 | May 14, 2018 | 0 |
| Accenture | https://www.accenture.com/t20180227T215953Z__w__/us-en/_acnmedia/Accenture/next-gen-7/tech-vision-2018/pdf/Accenture-TechVision-2018-Tech-Trends-Report.pdf | 1 | May 14, 2018 | 1 |
| Future of Humanities Institute, University of Oxford | https://www.fhi.ox.ac.uk/publications/ | 1 | May 14, 2018 | 1 |
| Centre for International Governance Innovation | https://www.cigionline.org/search?search_api_fulltext=A%20National%20Data%20&sort_by=search_api_relevance | 1 | May 14, 2018 | 1 |
| Access Now | https://www.accessnow.org/the-toronto-declaration-protecting-the-rights-to-equality-and-non-discrimination-in-machine-learning-systems/ | 1 | May 14, 2018 | 1 |
| UK Government | https://www.gov.uk/government/publications/code-of-conduct-for-data-driven-health-and-care-technology/initial-code-of-conduct-for-data-driven-health-and-care-technology | 1 | Sept 12, 2018 | 1 |
| British Standards Institution | https://drive.google.com/file/d/0B0McUk5vAotHcDYzbzh2Y0c4aUE/view | 1 | Sept 12, 2018 | 1 |
| Information Technology Industry Council | https://www.itic.org/public-policy/ITIAIPolicyPrinciplesFINAL.pdf | 1 | Sept 12, 2018 | 1 |
| Other (government websites, conferences, journal articles) | (Results from Environmental Scan) | 18 | April 25, 2018 | 16 |

*File name:* **Additional File 2**

*File format:* Word document (.docx)

*Title of data:* **Search Strategy and Results of Grey Literature Search**

*Description of data:* Search Strategy and Results of Grey Literature Search

**Grey Literature Databases** (May 10, 2018)

Total # Records before de-duplication: 22

Total # Records after de-duplication: 21

| Database | Search Strategy | # Records Screened | # New Potentially Relevant Records (after de | #Records after de-duplication |
| --- | --- | --- | --- | --- |
| Google Scholar | Artificial Intelligence and health and ethics | 50 | 20 | 19 |
| Des Lebris/Canadian Electronic Library | Artificial Intelligence | 4 | 0 | 0 |
| Canadian Institute for Health Information | Artificial Intelligence | 0 | 0 | 0 |
|  | Machine Learning | 5 | 0 | 0 |
| OAIster | Artificial Intelligence AND health AND ethics | 13 | 2 | 2 |

**Customized Google Search Engines** (May 13, 2018)

Total # Records before de-duplication: 26

Total # Records after de-duplication: 24

| Search Engine | Search Strategy | # Records Screened | | # New Potentially Relevant Records | #Records after de-duplication |
| --- | --- | --- | --- | --- | --- |
| Think Tanks | Artificial intelligence and health and ethics | 50 | | 7 | 7 |
| Non-Governmental Organizations | Artificial intelligence and health and ethics | 50 | | 0 | 0 |
| Canadian Government Documents | Artificial intelligence and health and ethics | Federal Documents | 0 | 0 | 0 |
|  |  | Provincial Documents | 0 | 0 | 0 |
|  |  | Municipal Documents | 0 | 0 | 0 |
|  |  | Depository Publications (Online Catalogue) | 4 | 2 | 2 |
| Google Search | Artificial intelligence and health and ethics | 45 | | 17 | 15 |

**Targeted Websites**

Total # Records before de-duplication: 37

Total # Records after de-duplication: 35

| Websites | URL | # Records Identified | Date Accessed | #Records after de-duplication |
| --- | --- | --- | --- | --- |
| MacArther Foundation | https://www.macfound.org/ | 0 | May 13, 2018 | 0 |
| Peterson Institute for International Economics | https://piie.com/ | 0 | May 13, 2018 | 0 |
| American Civil Liberties Union | https://www.aclu.org/ | 0 | May 13, 2018 | 0 |
| UC Berkeley | https://www.berkeley.edu/ | 0 | May 13, 2018 | 0 |
| Association for the Advancement of Artificial Intelligence at ASU | https://www.aaai.org/ | 0 | May 13, 2018 | 0 |
| OpenAI | https://openai.com/ | 0 | April 30, 2018 | 0 |
| Artificial Intelligence in Medicine Inc. | http://www.aim.ca/ | 0 | May 13, 2018 | 0 |
| AI Now Institute | https://ainowinstitute.org/ | 4 | May 13, 2018 | 4 |
| Mowat Centre | https://mowatcentre.ca/ | 0 | May 13, 2018 | 0 |
| Nuffield Council on Bioethics | http://nuffieldbioethics.org/ | 1 | April 25, 2018 | 1 |
| European Data Protection Supervisor - Ethics Advisory Group | https://royalsociety.org/~/media/policy/projects/data-governance/data-management-governance.pdf | 1 | April 25, 2018 | 1 |
| Stanford University | https://ai100.stanford.edu/2016-report | 1 | April 25, 2018 | 1 |
| The AI Initiative and The Future Society - Harvard Kennedy School | http://ai-initiative.org/wp-content/uploads/2017/08/Making-the-AI-Revolution-work-for-everyone.-Report-to-OECD.-MARCH-2017.pdf | 1 | April 25, 2018 | 1 |
| Centre for Internet and Society (CIS) | https://cis-india.org/internet-governance/files/ai-and-healtchare-report | 1 | April 25, 2018 | 1 |
| IEEE Global Initiative on Ethics of Autonomous and Intelligent Systems | https://standards.ieee.org/develop/indconn/ec/autonomous_systems.html | 1 | April 25, 2018 | 1 |
| The Alan Turing Institute | https://www.turing.ac.uk/ | 0 | May 14, 2018 | 0 |
| Canadian Institute for Advanced Research | https://www.cifar.ca/ai/ | 0 | May 14, 2018 | 0 |
| McKinsey | https://www.mckinsey.com/~/media/mckinsey/global%20themes/china/artificial%20intelligence%20implications%20for%20china/mgi-artificial-intelligence-implications-for-china.ashx | 1 | April 20, 2018 | 1 |
| Deloitte | https://www2.deloitte.com/insights/us/en/deloitte-review/issue-21/artificial-intelligence-and-the-future-of-work.html | 1 | May 14, 2018 | 1 |
| CBI | http://www.cbi.org.uk/about/about-us/ | 0 | May 14, 2018 | 0 |
| Vector Institute for Artificial Intelligence | https://vectorinstitute.ai/ | 0 | May 14, 2018 | 0 |
| Accenture | https://www.accenture.com/t20180227T215953Z__w__/us-en/_acnmedia/Accenture/next-gen-7/tech-vision-2018/pdf/Accenture-TechVision-2018-Tech-Trends-Report.pdf | 1 | May 14, 2018 | 1 |
| Future of Humanities Institute, University of Oxford | https://www.fhi.ox.ac.uk/publications/ | 1 | May 14, 2018 | 1 |
| Centre for International Governance Innovation | https://www.cigionline.org/search?search_api_fulltext=A%20National%20Data%20&sort_by=search_api_relevance | 1 | May 14, 2018 | 1 |
| Access Now | https://www.accessnow.org/the-toronto-declaration-protecting-the-rights-to-equality-and-non-discrimination-in-machine-learning-systems/ | 1 | May 14, 2018 | 1 |
| UK Government | https://www.gov.uk/government/publications/code-of-conduct-for-data-driven-health-and-care-technology/initial-code-of-conduct-for-data-driven-health-and-care-technology | 1 | Sept 12, 2018 | 1 |
| British Standards Institution | https://drive.google.com/file/d/0B0McUk5vAotHcDYzbzh2Y0c4aUE/view | 1 | Sept 12, 2018 | 1 |
| Information Technology Industry Council | https://www.itic.org/public-policy/ITIAIPolicyPrinciplesFINAL.pdf | 1 | Sept 12, 2018 | 1 |
| Other (government websites, conferences, journal articles) | (Results from Environmental Scan) | 18 | April 25, 2018 | 16 |
