# Supplementary material for: Artificial intelligence for good health: a scoping review of the ethics literature
Source: BMC Med Ethics. 2021 Feb 15;22:14. doi: 10.1186/s12910-021-00577-8 (PMC7885243; doi:10.1186/s12910-021-00577-8)

*File name:* **Additional File 3**

*File format:* Word document (.docx)

*Title of data:* **Data Charting Form Template**

*Description of data:* Template used to chart the data from the articles reviewed


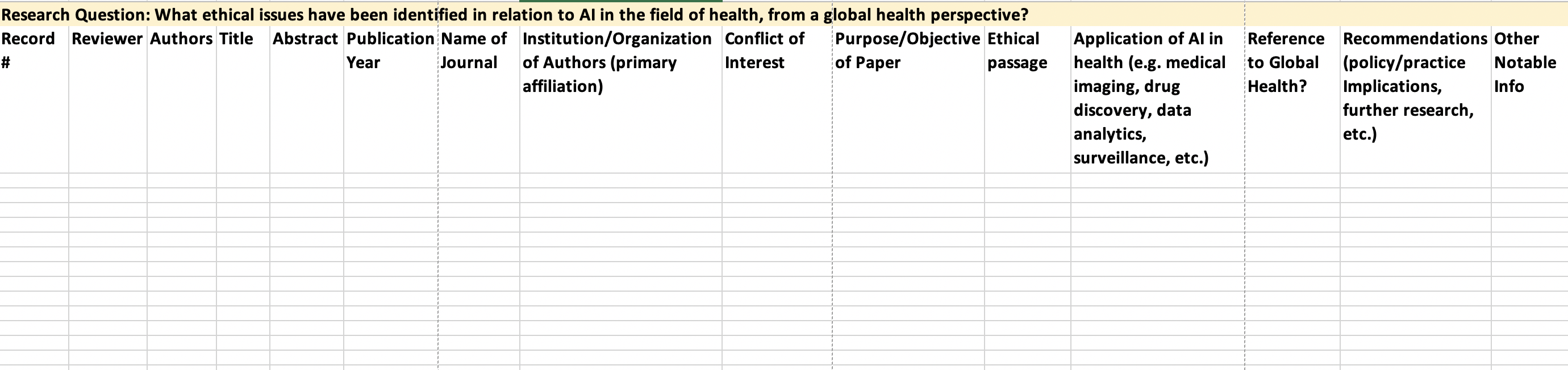

Supplement: Supplementary file 3 — Additional file 3. Data Charting Form Template. [file 12910_2021_577_MOESM3_ESM.docx]
