# Supplementary material for: Artificial intelligence for good health: a scoping review of the ethics literature
Source: BMC Med Ethics. 2021 Feb 15;22:14. doi: 10.1186/s12910-021-00577-8 (PMC7885243; doi:10.1186/s12910-021-00577-8)
Supplement: Supplementary file 4 — Additional file 4. Bibliography of the 103 records included in analysis. [file 12910_2021_577_MOESM4_ESM.docx]

*File name:* **Additional File 4**

*File format:* Word document (.docx)

*Title of data:* **Bibliography of the 103 records included in analysis**

*Description of data:* Below are the references for each recorded included in our analysis of the grey and academic literatures

1. Centre for International Governance Innovation. A national data strategy for Canada: Key elements and policy considerations [Internet]. 2018 p. 1–13. Report No.: 160. Available from: <https://www.cigionline.org/sites/default/files/documents/Paper%20no.160_3.pdf>

2. Coeckelbergh M, Pop C, Simut R, Peca A, Pintea S, David D, et al. A Survey of Expectations About the Role of Robots in Robot-Assisted Therapy for Children with ASD: Ethical Acceptability, Trust, Sociability, Appearance, and Attachment. Sci Eng Ethics [Internet]. 2016 Feb;22(1):47–65. Available from: <http://link.springer.com/10.1007/s11948-015-9649-x>

3. Mohandas S, Ranganathan R. AI and Healthcare in India: Looking Forward Roundtable Report. [Internet]. India: The Centre for Internet and Society, India; 2017. Available from: <https://cis-india.org/internet-governance/files/ai-and-healthcare-report>

4. International Telecommunications Union, XPrize. AI For Good Global Summit Report. AI For Good Global Summit [Internet]. Geneva, Switzerland; 2017. Available from: <https://www.itu.int/en/ITU-T/AI/Documents/Report/AI_for_Good_Global_Summit_Report_2017.pdf>

5. Infosys Limited. AI for Healthcare: Balancing Efficiency and Ethics. 2018;14. Available from: <https://www.infosys.com/smart-automation/docpdf/ai-healthcare.pdf>

6. AI in the UK: Ready, Willing and Able? [Internet]. United Kingdom: Authority of the House of Lords; 2018. (Intelligence SCoA, editor). Available from: <https://publications.parliament.uk/pa/ld201719/ldselect/ldai/100/100.pdf>

7. AI Now 2016 Symposium: The Social Implications of Artificial Intelligence Technologies in the Near-Term [Internet]. New York: AI Now; 2016. (AI Now 2016 Primers). Available from: <https://ainowinstitute.org/AI_Now_2016_Primers.pdf>

8. Campolo A, Sanfilippo M, Whittaker M, Crawford K. AI Now 2017 Report [Internet]. New York University; 2017. (AI Now 2017 Symposium and Workshop). Available from: <https://ainowinstitute.org/AI_Now_2017_Report.pdf>

9. Senders JT, Zaki MM, Karhade AV, Chang B, Gormley WB, Broekman ML, et al. An introduction and overview of machine learning in neurosurgical care. Acta Neurochir [Internet]. 2018 Jan;160(1):29–38. Available from: <http://link.springer.com/10.1007/s00701-017-3385-8>

10. Murphy J, Pardeck J. Application on the relevance of AI assumptions for social practitioners. Social Epistemology [Internet]. 1989 Oct;3(4):349–54. Available from: <http://www.tandfonline.com/doi/abs/10.1080/02691728908578547>

11. Hengstler M, Enkel E, Duelli S. Applied artificial intelligence and trust—The case of autonomous vehicles and medical assistance devices. Technological Forecasting and Social Change [Internet]. 2016 Apr;105:105–20. Available from: <https://linkinghub.elsevier.com/retrieve/pii/S0040162515004187>

12. Suominen H, Lehtikunnas T, Back B, Karsten H, Salakoski T, Salanterä S. Applying language technology to nursing documents: Pros and cons with a focus on ethics. International Journal of Medical Informatics [Internet]. 2007 Oct [cited 2020 Sep 23];76:S293–301. Available from: <https://linkinghub.elsevier.com/retrieve/pii/S1386505607001013>

13. Coeckelbergh M. Artificial agents, good care, and modernity. Theor Med Bioeth [Internet]. 2015 Aug;36(4):265–77. Available from: <http://link.springer.com/10.1007/s11017-015-9331-y>

14. Nuffield Council on Bioethics. 50. Artificial intelligence (AI) in healthcare and research [Internet]. 2018. Available from: <https://www.nuffieldbioethics.org/publications/ai-in-healthcare-and-research>

15. Stone P, Brooks R, Brynjolfsson E, Calo R, Etzioni O, Hager O, et al. “Artificial Intelligence and Life in 2030.” One Hundred Year Study on Artificial Intelligence: Report of the 2015-2016 Study Panel [Internet]. Available from: <https://ai100.stanford.edu/sites/g/files/sbiybj9861/f/ai_100_report_0831fnl.pdf>

16. Bonderman D. Artificial intelligence in cardiology. Wien Klin Wochenschr [Internet]. 2017 Dec;129(23–24):866–8. Available from: <http://link.springer.com/10.1007/s00508-017-1275-y>

17. Paul Y, Hickok E, Sinha A, Tiwari U, Bidare PM. Artificial Intelligence in the Healthcare Industry in India. 2018;45. Available from: <https://cis-india.org/internet-governance/files/ai-and-healtchare-report>

18. Kantarjian H, Yu PP. Artificial Intelligence, Big Data, and Cancer. JAMA Oncol [Internet]. 2015 Aug 1;1(5):573. Available from: <http://oncology.jamanetwork.com/article.aspx?doi=10.1001/jamaoncol.2015.1203>

19. Williams AM, Liu Y, Regner KR, Jotterand F, Liu P, Liang M. Artificial intelligence, physiological genomics, and precision medicine. Physiological Genomics [Internet]. 2018 Apr 1;50(4):237–43. Available from: <https://www.physiology.org/doi/10.1152/physiolgenomics.00119.2017>

20. Bowser A, Sloan M, Michelucci P, Pauwels E. Artificial intelligence: A policy-oriented introduction. [Internet]. Wilson Center; 2017. Available from: <https://www.wilsoncenter.org/sites/default/files/media/documents/publication/wilson_center_policy_brief_artificial_intelligence.pdf>

21. Monteith S, Glenn T. Automated Decision-Making and Big Data: Concerns for People With Mental Illness. Curr Psychiatry Rep [Internet]. 2016 Dec;18(12):112. Available from: <http://link.springer.com/10.1007/s11920-016-0746-6>

22. van der Plas A, Smits M, Wehrmann C. Beyond Speculative Robot Ethics: A Vision Assessment Study on the Future of the Robotic Caretaker. Accountability in Research [Internet]. 2010 Nov 18;17(6):299–315. Available from: <https://www.tandfonline.com/doi/full/10.1080/08989621.2010.524078>

23. Kruskal JB, Berkowitz S, Geis JR, Kim W, Nagy P, Dreyer K. Big Data and Machine Learning—Strategies for Driving This Bus: A Summary of the 2016 Intersociety Summer Conference. Journal of the American College of Radiology [Internet]. 2017 Jun;14(6):811–7. Available from: <https://linkinghub.elsevier.com/retrieve/pii/S1546144017301990>

24. Mooney SJ, Pejaver V. Big Data in Public Health: Terminology, Machine Learning, and Privacy. Annu Rev Public Health [Internet]. 2018 Apr;39(1):95–112. Available from: <http://www.annualreviews.org/doi/10.1146/annurev-publhealth-040617-014208>

25. Lustberg T, van Soest J, Jochems A, Deist T, van Wijk Y, Walsh S, et al. Big Data in radiation therapy: challenges and opportunities. BJR [Internet]. 2017 Jan;90(1069):20160689. Available from: <http://www.birpublications.org/doi/10.1259/bjr.20160689>

26. Boissoneault J, Sevel L, Letzen J, Robinson M, Staud R. Biomarkers for Musculoskeletal Pain Conditions: Use of Brain Imaging and Machine Learning. Curr Rheumatol Rep [Internet]. 2017 Jan;19(1):5. Available from: <http://link.springer.com/10.1007/s11926-017-0629-9>

27. Coeckelbergh M. Care robots and the future of ICT-mediated elderly care: a response to doom scenarios. AI & Soc [Internet]. 2016 Nov;31(4):455–62. Available from: <http://link.springer.com/10.1007/s00146-015-0626-3>

28. Vallor S. Carebots and Caregivers: Sustaining the Ethical Ideal of Care in the Twenty-First Century. Philos Technol [Internet]. 2011 Sep;24(3):251–68. Available from: <http://link.springer.com/10.1007/s13347-011-0015-x>

29. Decker M. Caregiving robots and ethical reflection: the perspective of interdisciplinary technology assessment. AI & Soc [Internet]. 2008 Jan;22(3):315–30. Available from: <http://link.springer.com/10.1007/s00146-007-0151-0>

30. Kenneth K, Eggleton A. Challenge ahead: Integrating robotics, artificial intelligence and 3D printing technologies into Canada’s healthcare systems. [Internet]. 2017 p. 1–44. (The Standing Senate Committee on Social Affairs SaT, editor). Available from: <https://sencanada.ca/content/sen/committee/421/SOCI/reports/RoboticsAI3DFinal_Web_e.pdf>

31. Hope M. Computer-aided medicine: present and future issues of liability. Computer-Law Journal. 1989;9(2):177–03.

32. Metzler TA, Lewis LM, Pope LC. Could robots become authentic companions in nursing care?: Robots Authentic Companions. Nursing Philosophy [Internet]. 2016 Jan;17(1):36–48. Available from: <http://doi.wiley.com/10.1111/nup.12101>

33. McBee MP, Awan OA, Colucci AT, Ghobadi CW, Kadom N, Kansagra AP, et al. Deep Learning in Radiology. Academic Radiology [Internet]. 2018 Nov;25(11):1472–80. Available from: <https://linkinghub.elsevier.com/retrieve/pii/S1076633218301041>

34. van Wynsberghe A. Designing Robots for Care: Care Centered Value-Sensitive Design. Sci Eng Ethics [Internet]. 2013 Jun;19(2):407–33. Available from: <http://link.springer.com/10.1007/s11948-011-9343-6>

35. Wu Y-H, Fassert C, Rigaud A-S. Designing robots for the elderly: Appearance issue and beyond. Archives of Gerontology and Geriatrics [Internet]. 2012 Jan [cited 2020 Sep 24];54(1):121–6. Available from: <https://linkinghub.elsevier.com/retrieve/pii/S0167494311000288>

36. Simpson RL. e-Ethics: New Dilemmas Emerge Alongside New Technologies. Nursing Administration Quarterly [Internet]. 2005 Apr;29(2):179–82. Available from: <http://journals.lww.com/00006216-200504000-00013>

37. Wolbring G, Diep L, Yumakulov S, Ball N, Leopatra V, Yergens D. Emerging Therapeutic Enhancement Enabling Health Technologies and Their Discourses: What Is Discussed within the Health Domain? Healthcare [Internet]. 2013 Jul 25 [cited 2020 Sep 24];1(1):20–52. Available from: <http://www.mdpi.com/2227-9032/1/1/20>

38. Körtner T. Ethical challenges in the use of social service robots for elderly people. Z Gerontol Geriat [Internet]. 2016 Jun;49(4):303–7. Available from: <http://link.springer.com/10.1007/s00391-016-1066-5>

39. Draper H, Sorell T. Ethical values and social care robots for older people: an international qualitative study. Ethics Inf Technol [Internet]. 2017 Mar [cited 2020 Sep 23];19(1):49–68. Available from: <http://link.springer.com/10.1007/s10676-016-9413-1>

40. Fenech M, Strukelj N, Buston O. Ethical, Social, and Political Challenges of Artificial Intelligence in Health [Internet]. Future Advocacy & Wellcome Trust; Available from: <https://wellcome.ac.uk/sites/default/files/ai-in-health-ethical-social-political-challenges.pdf>

41. Ethically Aligned Design: A Vision for Prioritizing Human Well-Being with Autonomous and Intelligent Systems [Internet]. The IEEE Global Initiative on Ethics of Autonomous and Intelligent Systems; n.d. Available from: <https://standards.ieee.org/content/dam/ieee-standards/standards/web/documents/other/ead1e.pdf>

42. Russell S. Ethics of artificial intelligence. 521:415–8. Available from: <https://www.nature.com/articles/521415a.pdf?origin=ppub>

43. Yuste R, Goering S, Arcas BA y, Bi G, Carmena JM, Carter A, et al. Four ethical priorities for neurotechnologies and AI. Nature [Internet]. 2017 Nov;551(7679):159–63. Available from: <http://www.nature.com/articles/551159a>

44. Corbett J, d’Angelo C, Gangitano L, Freeman J. Future of Health: Findings from a survey of stakeholders on the future of health and healthcare in England [Internet]. RAND Corporation; 2017 p. 1–90. Available from: <https://www.rand.org/pubs/research_reports/RR2147.html>

45. Powles J, Hodson H. Google DeepMind and healthcare in an age of algorithms. Health Technol [Internet]. 2017 Dec;7(4):351–67. Available from: <http://link.springer.com/10.1007/s12553-017-0179-1>

46. Sharkey A, Sharkey N. Granny and the robots: ethical issues in robot care for the elderly. Ethics Inf Technol [Internet]. 2012 Mar;14(1):27–40. Available from: <http://link.springer.com/10.1007/s10676-010-9234-6>

47. Moreno A. Guest Editor’s Introduction: On the Evolution of Applying Agent Technology to Healthcare. IEEE Intell Syst [Internet]. 2006 Nov;21(6):8–10. Available from: <http://ieeexplore.ieee.org/document/4042529/>

48. UK Government. Guidance: Initial code of conduct for data-driven health and care technology [Internet]. United Kingdom: Department of Health and Social Care, editor.; 2018. Available from: <https://www.gov.uk/government/publications/code-of-conduct-for-data-driven-health-and-care-technology/initial-code-of-conduct-for-data-driven-health-and-care-technology>

49. Coeckelbergh M. Health Care, Capabilities, and AI Assistive Technologies. Ethic Theory Moral Prac [Internet]. 2010 Apr;13(2):181–90. Available from: <http://link.springer.com/10.1007/s10677-009-9186-2>

50. Vandemeulebroucke T, de Casterlé BD, Gastmans C. How do older adults experience and perceive socially assistive robots in aged care: a systematic review of qualitative evidence. Aging & Mental Health [Internet]. 2018 Feb;22(2):149–67. Available from: <https://www.tandfonline.com/doi/full/10.1080/13607863.2017.1286455>

51. Johnson W, Pauwels E. How to Optimize Human Biology: :27. Available from: <https://www.wilsoncenter.org/sites/default/files/media/documents/publication/how_to_optimize_human_biology.pdf>

52. Char DS, Shah NH, Magnus D. Implementing Machine Learning in Health Care — Addressing Ethical Challenges. N Engl J Med [Internet]. 2018 Mar 15;378(11):981–3. Available from: <http://www.nejm.org/doi/10.1056/NEJMp1714229>

53. Kohli M, Prevedello LM, Filice RW, Geis JR. Implementing Machine Learning in Radiology Practice and Research. American Journal of Roentgenology [Internet]. 2017 Apr;208(4):754–60. Available from: <http://www.ajronline.org/doi/10.2214/AJR.16.17224>

54. Sparrow R, Sparrow L. In the hands of machines? The future of aged care. Minds & Machines [Internet]. 2006 Oct 18;16(2):141–61. Available from: <http://link.springer.com/10.1007/s11023-006-9030-6>

55. Ishihara K, Fukushi T. Introduction: Roboethics as an Emerging Field of Ethics of Technology. Accountability in Research [Internet]. 2010 Nov 18;17(6):273–7. Available from: <https://www.tandfonline.com/doi/full/10.1080/08989621.2010.523672>

56. Conner DA. Issues and techniques in networked-based distributed healthcare: Overview. Journal of Systems Integration [Internet]. 2000;10(1):81–94. Available from: <http://link.springer.com/10.1023/A:1026563402605>

57. Hodges BD. Learning from Dorothy Vaughan: artificial intelligence and the health professions. Med Educ [Internet]. 2018 Jan;52(1):11–3. Available from: <http://doi.wiley.com/10.1111/medu.13350>

58. Cheshire W. Loopthink: A limitation of medical artificial intelligence. Ethics and Medicine [Internet]. 2017;33(1):7–12. Available from: <https://www.scopus.com/record/display.uri?eid=2-s2.0-85010966212&origin=inward&txGid=e8aed5cfd7f105ac4743e546fa39ea4f>

59. Deng B. Machine ethics: The robot’s dilemma. Nature [Internet]. 2015 Jul;523(7558):24–6. Available from: <http://www.nature.com/articles/523024a>

60. Rudin C, Wagstaff KL. Machine learning for science and society. Mach Learn [Internet]. 2014 Apr;95(1):1–9. Available from: <http://link.springer.com/10.1007/s10994-013-5425-9>

61. Lee CH, Yoon H-J. Medical big data: promise and challenges. Kidney Res Clin Pract [Internet]. 2017 Mar 31;36(1):3–11. Available from: <http://www.krcp-ksn.org/journal/view.html?doi=10.23876/j.krcp.2017.36.1.3>

62. Mohammadzadeh N, Safdari R, Rahimi A. Multi-Agent System as a New Approach to Effective Chronic Heart Failure Management: Key Considerations. Healthc Inform Res [Internet]. 2013;19(3):162. Available from: <http://e-hir.org/journal/view.php?id=10.4258/hir.2013.19.3.162>

63. Mohammadzadeh N, Safdari R, Rahimi A. Multi-Agent Systems: Effective Approach for Cancer Care Information Management. Asian Pacific Journal of Cancer Prevention [Internet]. 2013 Dec 31 [cited 2020 Sep 24];14(12):7757–9. Available from: <http://koreascience.or.kr/journal/view.jsp?kj=POCPA9&py=2013&vnc=v14n12&sp=7757>

64. Ching T, Himmelstein DS, Beaulieu-Jones BK, Kalinin AA, Do BT, Way GP, et al. Opportunities and obstacles for deep learning in biology and medicine. J R Soc Interface [Internet]. 2018 Apr;15(141):20170387. Available from: <https://royalsocietypublishing.org/doi/10.1098/rsif.2017.0387>

65. Flahault A, Geissbuhler A, Guessous I, Guerin PJ, Bolon I, Marcel S, et al. Precision global health in the digital age. Swiss Med Wkly [Internet]. 2017 Apr 7;147(1314). Available from: <http://doi.emh.ch/smw.2017.14423>

66. Mentis A-FA, Pantelidi K, Dardiotis E, Hadjigeorgiou GM, Petinaki E. Precision Medicine and Global Health: The Good, the Bad, and the Ugly. Front Med [Internet]. 2018 Mar 14;5:67. Available from: <http://journal.frontiersin.org/article/10.3389/fmed.2018.00067/full>

67. Sacchi L, Holmes JH. Progress in Biomedical Knowledge Discovery: A 25-year Retrospective. Yearb Med Inform [Internet]. 2016 Aug;25(S 01):S117–29. Available from: <http://www.thieme-connect.de/DOI/DOI?10.15265/IYS-2016-s033>

68. Balthazar P, Harri P, Prater A, Safdar NM. Protecting Your Patients’ Interests in the Era of Big Data, Artificial Intelligence, and Predictive Analytics. Journal of the American College of Radiology [Internet]. 2018 Mar;15(3):580–6. Available from: <https://linkinghub.elsevier.com/retrieve/pii/S1546144017315995>

69. Markowetz A, Błaszkiewicz K, Montag C, Switala C, Schlaepfer TE. Psycho-Informatics: Big Data shaping modern psychometrics. Medical Hypotheses [Internet]. 2014 Apr;82(4):405–11. Available from: <https://linkinghub.elsevier.com/retrieve/pii/S0306987713005598>

70. Luxton DD. Recommendations for the ethical use and design of artificial intelligent care providers. Artificial Intelligence in Medicine [Internet]. 2014 Sep;62(1):1–10. Available from: <https://linkinghub.elsevier.com/retrieve/pii/S0933365714000682>

71. Albrecht S, Bouchard B, Brownstein JS, Buckeridge DL, Caragea C, Carter KM, et al. Reports of the 2016 AAAI Workshop Program. AIMag [Internet]. 2016 Oct 7;37(3):99. Available from: <https://aaai.org/ojs/index.php/aimagazine/article/view/2680>

72. Borenstein J, Pearson Y. Robot caregivers: harbingers of expanded freedom for all? Ethics Inf Technol [Internet]. 2010 Sep;12(3):277–88. Available from: <http://link.springer.com/10.1007/s10676-010-9236-4>

73. Sorell T, Draper H. Robot carers, ethics, and older people. Ethics Inf Technol [Internet]. 2014 Sep;16(3):183–95. Available from: <http://link.springer.com/10.1007/s10676-014-9344-7>

74. Siqueira-Batista R, Souza CR, Maia PM, Siqueira SL. ROBOTIC SURGERY: BIOETHICAL ASPECTS. ABCD, arq bras cir dig [Internet]. 2016 Dec;29(4):287–90. Available from: <http://www.scielo.br/scielo.php?script=sci_arttext&pid=S0102-67202016000400287&lng=en&tlng=en>

75. Holder C, Khurana V, Hook J, Bacon G, Day R. Robotics and law: Key legal and regulatory implications of the robotics age (part II of II). Computer Law & Security Review [Internet]. 2016 Aug;32(4):557–76. Available from: <https://linkinghub.elsevier.com/retrieve/pii/S0267364916300899>

76. Sharkey A. Robots and human dignity: a consideration of the effects of robot care on the dignity of older people. Ethics Inf Technol [Internet]. 2014 Mar;16(1):63–75. Available from: <http://link.springer.com/10.1007/s10676-014-9338-5>

77. O’Brolcháin F. Robots and people with dementia: Unintended consequences and moral hazard. Nurs Ethics [Internet]. 2019 Jun;26(4):962–72. Available from: <http://journals.sagepub.com/doi/10.1177/0969733017742960>

78. van Kemenade M, Konijn EA, Hoorn J. Robots Humanize Care - Moral Concerns Versus Witnessed Benefits for the Elderly: In: Proceedings of the International Conference on Health Informatics [Internet]. Lisbon, Portugal: SCITEPRESS - Science and and Technology Publications; 2015. p. 648–53. Available from: <http://www.scitepress.org/DigitalLibrary/Link.aspx?doi=10.5220/0005287706480653>

79. Gallagher A, Nåden D, Karterud D. Robots in elder care: Some ethical questions. Nurs Ethics [Internet]. 2016 Jun;23(4):369–71. Available from: <http://journals.sagepub.com/doi/10.1177/0969733016647297>

80. Dahl T, Boulos M. Robots in Health and Social Care: A Complementary Technology to Home Care and Telehealthcare? Robotics [Internet]. 2013 Dec 30;3(1):1–21. Available from: <http://www.mdpi.com/2218-6581/3/1/1>

81. Wachsmuth I. Robots Like Me: Challenges and Ethical Issues in Aged Care. Front Psychol [Internet]. 2018 Apr 3;9:432. Available from: <http://journal.frontiersin.org/article/10.3389/fpsyg.2018.00432/full>

82. van Wynsberghe A. Service robots, care ethics, and design. Ethics Inf Technol [Internet]. 2016 Dec;18(4):311–21. Available from: <http://link.springer.com/10.1007/s10676-016-9409-x>

83. Gentry T. Smart homes for people with neurological disability: State of the art. Zasler N, editor. NRE [Internet]. 2009 Oct 28;25(3):209–17. Available from: <https://www.medra.org/servlet/aliasResolver?alias=iospress&doi=10.3233/NRE-2009-0517>

84. Conway M, O’Connor D. Social media, big data, and mental health: current advances and ethical implications. Current Opinion in Psychology [Internet]. 2016 Jun;9:77–82. Available from: <https://linkinghub.elsevier.com/retrieve/pii/S2352250X16000063>

85. Laitinen A, Niemelä M, Pirhonen J. Social robotics, elderly care, and human dignity: A recognition-theoretical approach. In: What Social Robots Can and Should Do [Internet]. IOS Press; 2016. Available from: <https://doi.org/10.3233/978-1-61499-708-5-155>

86. Miner AS, Milstein A, Hancock JT. Talking to Machines About Personal Mental Health Problems. JAMA [Internet]. 2017 Oct 3;318(13):1217. Available from: <http://jama.jamanetwork.com/article.aspx?doi=10.1001/jama.2017.14151>

87. Pilotto A, Boi R, Petermans J. Technology in geriatrics. Age and Ageing [Internet]. 2018 Nov 1;47(6):771–4. Available from: <https://academic.oup.com/ageing/article/47/6/771/4931215>

88. Crawford K, Whittaker M. The AI Now Report: The social and economic implications of artificial intelligence technologies in the near-term. White House and New York University’s Information Law Institute; (AI Now Public Symposium 2016).

89. Sharts-Hopko NC. The Coming Revolution in Personal Care Robotics: What Does It Mean for Nurses? Nursing Administration Quarterly [Internet]. 2014;38(1):5–12. Available from: <http://journals.lww.com/00006216-201401000-00003>

90. Sharkey N, Sharkey A. The Eldercare Factory. Gerontology [Internet]. 2012;58(3):282–8. Available from: <https://www.karger.com/Article/FullText/329483>

91. Bollier D. The promise and challenge of integrating AI into cars. healthcare and journalism: A Report on the Inaugural Aspen Institute Roundtable on Artificial Intelligence [Internet]. United States: The Aspen Institute; 2017. Available from: <https://assets.aspeninstitute.org/content/uploads/2017/01/2017-Artificial-Intelligence-REP-FINAL.pdf>

92. Mesko B. The role of artificial intelligence in precision medicine. Expert Review of Precision Medicine and Drug Development [Internet]. 2017 Sep 3;2(5):239–41. Available from: <https://www.tandfonline.com/doi/full/10.1080/23808993.2017.1380516>

93. Iosa M, Morone G, Cherubini A, Paolucci S. The Three Laws of Neurorobotics: A Review on What Neurorehabilitation Robots Should Do for Patients and Clinicians. J Med Biol Eng [Internet]. 2016 Feb;36(1):1–11. Available from: <http://link.springer.com/10.1007/s40846-016-0115-2>

94. Howard A, Borenstein J. The Ugly Truth About Ourselves and Our Robot Creations: The Problem of Bias and Social Inequity. Sci Eng Ethics [Internet]. 2018 Oct;24(5):1521–36. Available from: <http://link.springer.com/10.1007/s11948-017-9975-2>

95. Vandemeulebroucke T, Dierckx de Casterlé B, Gastmans C. The use of care robots in aged care: A systematic review of argument-based ethics literature. Archives of Gerontology and Geriatrics [Internet]. 2018 Jan;74:15–25. Available from: <https://linkinghub.elsevier.com/retrieve/pii/S0167494317302790>

96. Huschilt J, Clune L. The Use of Socially Assistive Robots for Dementia Care. J Gerontol Nurs [Internet]. 2012 Oct 1;38(10):15–9. Available from: <http://www.healio.com/doiresolver?doi=10.3928/00989134-20120911-02>

97. Denton S, Pauwels E, He Y, Johson W. There’s nowhere to hide: Artificial intelligence and privacy in the fourth industrial revolution [Internet]. Wilson Center, Synenergene, and the Institute for Philosophy & Public Policy; 2018. Available from: <https://iapp.org/media/pdf/resource_center/ai_and_privacy.pdf>

98. Cios KJ, William Moore G. Uniqueness of medical data mining. Artificial Intelligence in Medicine [Internet]. 2002 Sep [cited 2020 Sep 24];26(1–2):1–24. Available from: <https://linkinghub.elsevier.com/retrieve/pii/S0933365702000490>

99. Vogel L. What “learning” machines will mean for medicine. CMAJ [Internet]. 2017 Apr 24;189(16):E615–6. Available from: <http://www.cmaj.ca/lookup/doi/10.1503/cmaj.1095413>

100. Bedaf S, Marti P, De Witte L. What are the preferred characteristics of a service robot for the elderly? A multi-country focus group study with older adults and caregivers. Assistive Technology [Internet]. 2019 May 27;31(3):147–57. Available from: <https://www.tandfonline.com/doi/full/10.1080/10400435.2017.1402390>

101. West DM. What happens if robots take the jobs? The impact of emerging technologies on employment and public policy [Internet]. Center for Technology Innovation at Brookings; 2015. Available from: <https://www.brookings.edu/wp-content/uploads/2016/06/robotwork.pdf>

102. Verghese A, Shah NH, Harrington RA. What This Computer Needs Is a Physician: Humanism and Artificial Intelligence. JAMA [Internet]. 2018 Jan 2;319(1):19. Available from: <http://jama.jamanetwork.com/article.aspx?doi=10.1001/jama.2017.19198>

103. Santoni de Sio F, van Wynsberghe A. When Should We Use Care Robots? The Nature-of-Activities Approach. Sci Eng Ethics [Internet]. 2016 Dec;22(6):1745–60. Available from: <http://link.springer.com/10.1007/s11948-015-9715-4>
